# Efficacy of genetically modified *Mycoplasma hyopneumoniae* strains and their effect on local and cell-mediated immune responses in pigs

**DOI:** 10.1186/s13567-025-01653-2

**Published:** 2025-11-17

**Authors:** Lisa Beuckelaere, Filip Boyen, Gaël Auray, Maarten Haspeslagh, Eva De Coensel, Bettina Salome Trueeb, Evelyne Meyer, Freddy Haesebrouck, Ward De Spiegelaere, Bert Devriendt, Artur Summerfield, Peter Kuhnert, Dominiek Maes

**Affiliations:** 1 Department of Internal Medicine, Reproduction and Population Medicine, Faculty of Veterinary Medicine, Ghent University, Merelbeke, Belgium; 2 Department of Pathobiology, Pharmacology and Zoological Medicine, Faculty of Veterinary Medicine, Ghent University, Merelbeke, Belgium; 3 Institute of Virology and Immunology, Mittelhäusern, Switzerland; 4 Department of Infectious Diseases and Pathobiology, Vetsuisse Faculty, University of Bern, Bern, Switzerland; 5 Department of Large Animal Surgery, Anaesthesia and Orthopaedics, Faculty of Veterinary Medicine, Ghent University, Merelbeke, Belgium; 6 Institute of Veterinary Bacteriology, Vetsuisse Faculty, University of Bern, Bern, Switzerland; 7 Department of Veterinary and Biosciences, Faculty of Veterinary Medicine, Ghent University, Merelbeke, Belgium; 8 Department of Morphology, Imaging, Orthopedics, Rehabilitation and Nutrition, Faculty of Veterinary Medicine, Ghent University, Merelbeke, Belgium; 9 Ghent University Digital PCR Center (DIGPCR), Ghent University, Ghent, Belgium; 10 Department of Translational Physiology, Infectiology and Public Health, Faculty of Veterinary Medicine, Ghent University, Merelbeke, Belgium

**Keywords:** *Mycoplasma hyopneumoniae*, enzootic pneumonia, vaccine efficacy, genetically modified *M. hyopneumoniae* strains, cell-mediated immune responses, pigs, *mmsA*, *mnuA*

## Abstract

Vaccination against *Mycoplasma hyopneumoniae* is still carried out worldwide, but unfortunately current commercial vaccines only provide partial protection. Therefore, two *M. hyopneumoniae* strains were genetically modified by transposon-mediated gene disruption of *mmsA* and *mnuA*, encoding methylmalonate semialdehyde dehydrogenase and membrane nuclease A, respectively. We investigated how immune responses elicited by these genetically modified *M. hyopneumoniae* strains protected pigs against challenge infection. An endotracheal single dose vaccination with genetically modified *M. hyopneumoniae* strain 1 (Δ*mmsA*) or 2 (Δ*mnuA*), or physiological saline solution (Control) was followed by challenge infection. Piglets from Δ*mnuA* had a higher respiratory disease score post-vaccination, but this group coughed significantly less after challenge. Significantly fewer DNA copies of the challenge strains were observed in broncho-alveolar lavage fluid (BAL) from Δ*mnuA* after challenge. Two weeks post-challenge, significantly more BAL IgG and BAL IgA was observed in Δ*mnuA*, but at euthanasia significantly more IgA and less pro-inflammatory cytokines were detected in BAL from both vaccinated groups. Furthermore, a significantly lower percentage of IFN-γ^+^ and TNF-α^+^IFN-γ^+^ CD8^+^ T cells was observed after administration of Δ*mnuA.* The percentage of IFN-γ^+^ CD8^+^ T cells was significantly lower in Δ*mmsA* at euthanasia. To conclude, the results of this exploratory study show that a single endotracheal administration of Δ*mnuA* resulted in coughing post-vaccination, but reduced clinical signs post-challenge and challenge strain DNA load in BAL. Therefore, a strain mutated in the *mnuA* gene might be an interesting mutant strain that could be promising as a potential live vaccine candidate strain as it can reduce *M. hyopneumoniae* infection burden under field conditions.

**Supplementary Information:**

The online version contains supplementary material available at 10.1186/s13567-025-01653-2.

## Introduction

*Mycoplasma hyopneumoniae* (homotypic synonym of *Mesomycoplasma hyopneumoniae*) is one of the most important respiratory pathogens associated with intensive pig production. This bacterium is known as the primary pathogen of enzootic pneumonia (EP), a chronic respiratory disease which mainly affects growing and fattening pigs [1].

Mycoplasmas like *M. hyopneumoniae* need to acquire essential nutrients to survive and proliferate in the host. These nutrients are either obtained from the host or synthesized de novo from basic components. One of the precursor molecules for phospholipids, which are essential for membrane integrity and intracellular signaling in eukaryotes and mycobacteria, is myo-inositol [2]. Myo-inositol is readily abundant in the bloodstream of mammalian hosts and it can be used as a secondary carbon source for energy production by bacteria in the extremely vascularized respiratory system [2, 3]. Interestingly, *M. hyopneumoniae* is so far the only reported species among the Mollicutes that contains genes involved in the uptake and catabolism of myo-inositol [3, 4]. Moreover, a previous transcriptome profiling of *M. hyopneumoniae* showed that all genes needed for myo-inositol catabolism were transcribed under normal culture conditions [3]. One of the genes involved in the myo-inositol pathway and present in the genome of *M. hyopneumoniae* is the gene encoding for methylmalonate semialdehyde dehydrogenase (MmsA) [5]. This bifunctional enzyme is important in one of the final steps of the myo-inositol metabolism as it catalyzes the NAD^+^-dependent oxidation of methylmalonate semialdehyde and malonate semialdehyde into propionyl-CoA and acetyl-CoA, respectively, twice with bicarbonate production [6]. The ability of *M. hyopneumoniae* to uptake and process host myo-inositol might explain why this *Mycoplasma* species can persist longer in the lungs as compared to other mycoplasmas, like *M. flocculare* or *M. hyorhinis* [3].

Mycoplasmas lack the biosynthetic capacity to synthesize de novo nucleic acid precursors [7]. These organisms thus need to scavenge nucleotides from their environment to survive in the host [8]. To obtain the necessary nucleotides, several *Mycoplasma* species, including *M. bovis* and *M. hyopneumoniae*, possess potent membrane nucleases, such as membrane nuclease A (MnuA) [9, 10]. MnuA can degrade DNA in neutrophil extracellular traps (NETs) [11]. Evading innate immunity by degrading NETs in a MnuA-dependent manner and using the released nucleotides for their own benefit is a survival strategy of mycoplasmas [12].

The main clinical sign of an infection with *M. hyopneumoniae* is a chronic, non-productive cough, but the disease also leads to performance losses, such as increased feed conversion, and elevated treatment costs [13, 14]. Improvement of management practices, use of antimicrobials, and vaccination are the most frequently applied measures to control *M. hyopneumoniae* infections [13]. Most commercial *M. hyopneumoniae* vaccines consist of inactivated, adjuvanted whole-cell preparations [13, 15, 16]. In addition, one vaccine based on only soluble antigens of *M. hyopneumoniae* [17] and two attenuated vaccines have been licensed (in China [18] and in Mexico (Vaxsafe^®^ MHP, Avimex) [19, 20]). Although current commercial vaccines against *M. hyopneumoniae* usually improve performance parameters and reduce clinical signs and lung lesions [15, 21–23], only partial protection is obtained and transmission of the pathogen cannot be prevented [24–26]. Most commercial *M. hyopneumoniae* vaccines are administered intramuscularly. It has been shown that (the quality of) the vaccine-induced immune responses can be affected by the administration route [27, 28]. For example, secretory IgA is typically not induced by parenteral vaccination [29], whereas mucosal attenuated vaccines might be able to induce secretory IgA. Secretory IgA is present at mucosal surfaces, including the mucus layer of the respiratory tract, and plays an important role in mucosal immunity as it can prevent pathogens such as *M. hyopneumoniae* from attaching to and penetrating host cells [30].

Extracellular bacteria and fungi are known to induce a type 3 immunity, which is associated with a strong Th17 response [29] and considered important for the protection of mucosal surfaces, as it promotes epithelial cell regeneration, mucous and antimicrobial protein production and neutrophil recruitment [31]. Immune responses needed to protect the host against a *M. hyopneumoniae* infection are not fully known yet, but it has been suggested that mucosal antibodies and cell-mediated immune responses, such as Th1 and Th17 responses, are important [29, 32].

Improvement of the current vaccines is needed not only to further reduce *M. hyopneumoniae*-induced clinical signs and economic losses, but also to combat pathogen transmission and colonization. Most commercial *M. hyopneumoniae* vaccines are inactivated vaccines based on the J strain, which might be less relevant for current circulating field strains as this strain was isolated in 1963 from a field outbreak in sows experiencing a mild course of enzootic pneumonia [33]. Therefore, using transposon mutagenesis we disrupted the *mnuA* and *mmsA* genes to attenuate the highly virulent *M. hyopneumoniae* strain F7.2C. The F7.2C strain was isolated in Belgium in 2000 from a pig with typical EP lesions at slaughter [34]. Attenuated vaccines might be key for the prevention of a *M. hyopneumoniae* infection because they are better at stimulating immune responses compared to inactivated vaccines [16, 35]. To increase mucosal immune responses, these genetically modified *M. hyopneumoniae* strains were administered endotracheally to pigs. In this study, we investigated the ability of these genetically modified *M. hyopneumoniae* strains to elicit antibody and T cell responses and to protect pigs against *M. hyopneumoniae* challenge infection.

## Materials and methods

### Attenuation of *M. hyopneumoniae* strain F7.2C via transposon gene disruption

A Tn-mutant library of *M. hyopneumoniae* strain F7.2C [36] was screened for specific Tn-disrupted genes using PCR. The mutant library was kept in multiple 96-well plates containing liquid medium (Mycoplasma Experience Ltd, Surrey, UK) supplemented with 0.3 µg/mL tetracycline. The library was replicated using 195 µL of medium and 5 µL of individual mutants and grown in wet chambers at 37 °C until the color of the medium changed from red to yellow. First, pools from plates were screened and then, from positive pools, single mutants were identified from corresponding plates.

Since the insertion direction of the transposon was unknown, forward 5’ located and reverse 3’ located primers were designed for each gene as shown in Table 1. Each of these primers was used in combination with the Tn-located primer NGS_1st_Seq6 allowing amplification of the specific gene insertion region. PCR was done in a total volume of 60 µL containing 1 × HOT FIREPol^®^ Master Mix Ready to load (Solis Biodyne, Tartu, Estonia), 0.4 µM of each primer, 6 µL DNA of the pooled or single mutants and filled up with ddH_2_O to the corresponding volume. The following cycle conditions were used for gene amplification: 95 °C 12 min followed by 35 cycles of 95 °C 30 s, 49 °C 30 s, 72 °C 90 s and a final elongation step of 72 °C 5 min. The resulting PCR products were Sanger sequenced (Microsynth AG, Balgach, Switzerland) using the same primers as for PCR to confirm and identify the gene-specific Tn-insertion site. After final identification of the wanted single mutants, they were purified as single colonies on agar plates (Mycoplasma Experience Ltd) containing 0.3 µg/mL tetracycline. Single colonies of Δ*mmsA* and Δ*mnuA* mutant strains were grown in the corresponding liquid medium and stored at −80 °C.

**Table 1:** **Primers and probes used for specific Tn-mutant screening and dPCR triplex assay**

PCR	Primer name	Sequence 5’—3’
Tn-mutant screening	NGS_1st_Seq6	GAC TTG AGC GTC GAT TTT TGT G
	Δ*mmsA*_ATG-1	GAA GCA GTT GCA GAG GTT GA
	Δ*mmsA*_TAA-1	GGC CAA ACC AGA ATC CTT C
	Δ*mnuA*_TAA-1	TGA CTG TTT TGG CCT AAT TCA A
	Δ*mnuA*_ATG-1	GGT TCT TTA GCC ACC GGA CT
dPCR triplex assay	Δ*mmsA* Fw	CTC AGA AGA TGC AGT TTC AAT TGG
	Δ*mmsA* Rv	TTG CGG TAC CCT TTT ACA CAA TTA
	Δ*mmsA* probe	GAG CAA GGA TAA AGT CCG
	Δ*mnuA* Fw	CGC AGA AGC TCG TGA TTT GA
	Δ*mnuA* Rv	ACG GTT GCG GTA CCC TTT TA
	Δ*mnuA* probe	AGA TAA AGT CCG TAT AAT TG
	P102 Fw	GTC AAA GTC AAA GTC AGC AAA C
	P102 Rv	AGC TGT TCA AAT GCT TGT CC
	P102 probe	ACC AGT TTC CAC TTC ATC GCC TCA

Tn-mutant screening: Forward 5’ located and reverse 3’ located primers were designed for each gene and each primer was used in combination with the Tn-located primer NGS_1st_Seq6 allowing amplification of the specific gene insertion region. These primers were used to check the insertion direction of the transposon and for Sanger sequencing (Microsynth) of the resulting PCR products to confirm and identify gene-specific Tn-insertion site.

dPCR triplex assay: TaqMan^™^ MGB hydrolysis probes targeting the Tn-insertion site and specific primers were designed to allow determination of the DNA copy number of each genetically modified *M. hyopneumoniae* strain separately. The total copy number of *M. hyopneumoniae* DNA was measured using the primers and probe designed by Marois et al. [47] in a recently developed dPCR assay [23] used to target the P102 gene of *M. hyopneumoniae*. The number of copies of both vaccine candidate strains was subtracted from the total copy number of *M. hyopneumoniae* DNA in order to determine the number of DNA copies of the challenge strains.

### Growth of mutant strains

The time required to grow the two *M. hyopneumoniae* mutant strains in modified liquid Friis medium was not known yet. Thus, the Δ*mmsA*, Δ*mnuA* and wild type F7.2C strains were grown several times and their growth kinetics were assessed using an ATP-dependent luminometric assay as described before [37]. After the pre-experimental growth tests, both genetically modified *M. hyopneumoniae* strains were grown until their peak ATP amount was reached in order to obtain the final inoculum of each genetically modified *M. hyopneumoniae* strain. To understand how ATP levels correlated with CCU for these strains, the concentration of both inocula was also determined using the CCU method [37].

### Animals and experimental design

Since *M. hyopneumoniae* only causes disease in pigs, this was the only animal species considered fit for the purpose of this study. This study is in compliance with the European Directive 2010/63/EU, and was approved by the Ethics Committee of the Faculty of Veterinary Medicine and the Faculty of Bioscience Engineering, Ghent University (EC 2018-52).

Forty-one *M. hyopneumoniae*-free Rattlerow-Seghers piglets (RA-SE Genetics NV, Ooigem, Belgium) were purchased from a herd that was free of *M. hyopneumoniae* and porcine reproductive and respiratory syndrome (PRRS) virus for several years based on repeated serological tests, and absence of clinical signs and lung lesions at slaughter. Twenty tracheobronchial swabs (TBS) were collected before the start of the experiment to test for the presence of *M. hyopneumoniae* using nested PCR [38] and all samples were negative.

**Figure 1:**
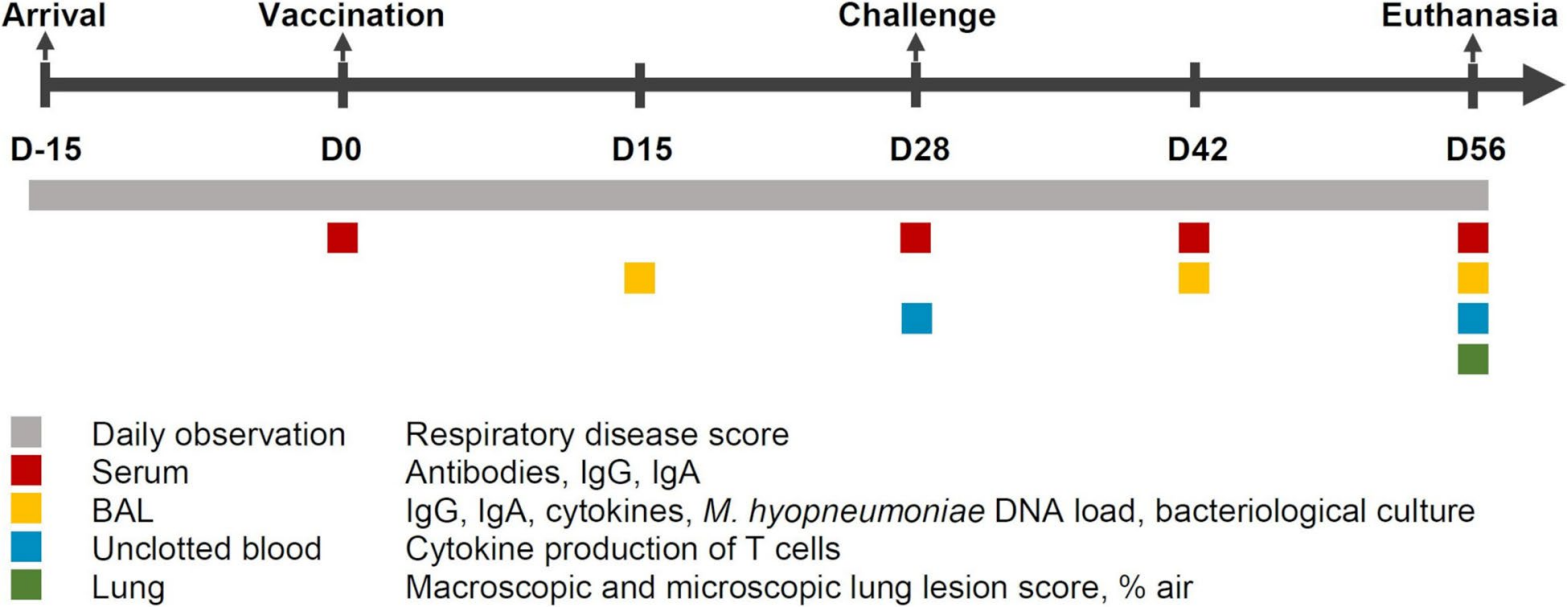
**Study design.** *Mycoplasma hyopneumoniae*-free piglets were randomly assigned to one of the following groups: non-vaccinated and non-challenged sentinel group (Sentinel; *n* = 5); non-vaccinated and challenged control group (Control; *n* = 12), vaccinated with genetically modified *M. hyopneumoniae* strain 1 and challenged group (Δ*mmsA*; *n* = 12); vaccinated with genetically modified *M. hyopneumoniae* strain 2 and challenged group (Δ*mnuA*; *n* = 12). BAL = broncho-alveolar lavage fluid; D = days.

An overview of the study and all samples collected is shown in Figure 1. Immediately after weaning, at approximately 28 days of age (D-15), piglets were transported to the experimental facilities and randomly allocated to one of four different groups: non-vaccinated and non-challenged sentinel group (Sentinel; *n* = 5), non-vaccinated and challenged control group (Control; *n* = 12), vaccinated with genetically modified *M. hyopneumoniae* strain 1 and challenged group (Δ*mmsA*; *n* = 12), and vaccinated with genetically modified *M. hyopneumoniae* strain 2 and challenged group (Δ*mnuA*; *n* = 12). Random numbers were generated using the standard = RAND() function in Microsoft Excel and each group had a similar average weight at arrival and the groups were gender balanced.

The main parameter used for the sample size calculation was the macroscopic lung lesion score, which ranged from 0 (no EP-like lesions) to 35 (entire lung affected) [39]. The presence of 12 animals in each treatment group allowed the detection of a biologically relevant difference in lung lesion score of 4.3 points with a standard deviation of 3.56 (two-sided test) with 95% certainty and 80% power. The sentinel group was used to verify whether the piglets remained negative for *M. hyopneumoniae* throughout the study. This group was not included in the statistical analyses, therefore fewer animals were required in this group. Power and sample size calculations were performed using SAS Power and Sample Size application (SAS Institute Inc., Cary, North Carolina, US) and was based on previous experimental studies performed by our research group [21, 22, 40].

Each group was housed in a similar but separate HEPA-filtered room for which relative humidity and temperature were measured daily using Gemini Tinytag Plus 2 data loggers (Gemini Data Loggers Ltd., Chichester, UK). Piglets had free access to drinking water and were fed ad libitum with a commercial diet without antimicrobials (Leievoeders N.V., Waregem, Belgium). Mild diarrhea was observed in all groups during the acclimation period, which led to the oral treatment of all animals with colistin sulphate (Colivet^®^ SF 500, Prodivet, Eynatten, Belgium) for 5 days, namely from D-14 till D-10 (dose 50 000 IU/kg body weight).

After an acclimatization period of 15 days, animals were anesthetized via intramuscular injection of 0.22 mL/kg body weight of a mixture of zolazepam and tiletamine (Zoletil 100^®^, Virbac, Louvain la Neuve, Belgium) and xylazine (Xyl-M^®^ 2%, VMD, Arendonk, Belgium) (D0). Next, 7 mL of sterile modified Friis medium was administered endotracheally to piglets from Sentinel and Control, while piglets from Δ*mmsA* and Δ*mnuA* were vaccinated by endotracheal inoculation of 7 mL modified Friis medium containing 583 pmol ATP/mL of Δ*mmsA* or 715 pmol ATP/mL of Δ*mnuA*, respectively.

Four weeks after vaccination (D28), piglets were anesthetized again to allow endotracheal inoculation of the animals from Δ*mmsA*, Δ*mnuA* and Control with 7 mL modified Friis medium containing 5 × 10^8^ CCU/mL of the high virulence strain *M. hyopneumoniae* F7.2C. The next day (D29), 7 mL modified Friis medium containing 5 × 10^8^ CCU/mL of a low virulence *M. hyopneumoniae* strain F1.12A was administered endotracheally to the piglets from these groups. For this infection model, two genetically different field strains were used, as this might improve extrapolation of the obtained results to the field situation since most pigs are simultaneously infected with two or more genetically different *M. hyopneumoniae* strains under field conditions [22]. Sterile modified Friis medium (7 mL) was administered on both challenge days to piglets from Sentinel. Four weeks after challenge (D56), all animals were euthanized by exsanguination after deep anesthesia by intramuscular injection of 0.3 mL/kg of the mixture of zolazepam, tiletamine and xylazine.

### Sample collection

Due to the risk of transferring *M. hyopneumoniae* to the non-vaccinated and non-challenged sentinel group, the experimenter could not be blinded during the animal experiment and sample collection, but laboratory and statistical analyses were performed in a blinded manner.

Serum samples were collected before vaccination (D0), before challenge (D28), 2 weeks post-challenge (D42) and at euthanasia (D56). Serum was stored at −20 °C until further analyses. Additionally, non-clotted blood was collected in EDTA tubes before challenge (D28) and at euthanasia (D56) and processed immediately to determine the effect of vaccination and subsequent challenge infection on the cell-mediated immune responses.

Two weeks post-vaccination (D15) and two weeks post-challenge (D42), broncho-alveolar lavage fluid (BAL) samples were collected by inserting a catheter (Portex^®^ Dog Catheter with Female Luer Mount, Smiths Medical International Ltd., Kent, UK) in the trachea which was used to flush the lungs with 20 mL sterile phosphate buffered saline (PBS) [22]. BAL samples were also collected immediately after euthanasia by flushing the head bronchus of the left part of the lung with 20 mL of sterile PBS [41]. Upon sample collection, a standard bacteriological examination was performed on the BAL samples from D56, as described below. All BAL samples were stored at −80 °C until further analyses.

### Clinical parameters

Piglets were observed daily by the same investigator for at least 20 min from D-15 until D56 between 8 and 10 a.m., and abnormal clinical findings, such as loss of appetite, diarrhea, dyspnea, tachypnea, depression, and lameness were recorded for each individual piglet. Furthermore, a daily respiratory disease score (RDS) was used to assess the severity of coughing for each individual animal [42]. The RDS ranged from 0 to 6: 0 = no coughing, 1 = mild coughing after an encouraged move, 2 = mild coughing in rest, 3 = moderate coughing after an encouraged move, 4 = moderate coughing in rest, 5 = severe coughing after an encouraged move, 6 = severe coughing in rest. The average RDS was calculated for each group after vaccination (from D0–D27) and after challenge (from D28–D56).

### Macroscopic and microscopic lung lesions

Immediately after exsanguination, the lungs were removed from the carcasses and scored for macroscopic EP-like lung lesions [39]. The macroscopic lung lesion score ranged from 0 (no typical EP-like lesions) to 35 (entire lung affected). Subsequently, a sample was collected for histopathological examination from the right apical, cardiac and diaphragmatic lung lobe from the border of the lesion (if present). Lung tissue samples were first fixed in 10% neutral buffered formalin and routinely processed and embedded in paraffin before staining with hematoxylin and eosin. Next, microscopic slides were scanned using a Nanozoomer NDP slide scanner (Hamamatsu Photonics, Hamamatsu, Japan) and its viewing platform (NDP.View2). The degree of peribronchiolar and perivascular lymphohistiocytic infiltration and nodule formation (cuffing) was assessed for 10 microscopic fields (multiplication 10x) of each sample using a previously described scoring system [34]. This microscopic lung lesion score ranged from 1 to 5, with 1 = limited infiltration of macrophages and lymphocytes around bronchioles, with airways and alveolar spaces free of cellular exudates, 2 = light to moderate infiltrates with mild diffuse cellular exudates into airways, 3–4–5 = (mild, moderate and severe, respectively) lesions characteristic of broncho-interstitial pneumonia, centered around bronchioles but extending to the interstitium, with lymphofollicular infiltration and mixed inflammatory cell exudates. Scores 3, 4 and 5 are suggestive for an infection with *M. hyopneumoniae*. The median microscopic lung lesion score was determined for each animal, and the percentage of lung area occupied by air (% air) was determined by analyzing 10 microscopic fields per sample with ImageJ (Bethesda Softworks, Rockville, MD, USA). The percentage of lung area occupied by air is inversely proportional to the lymphohistiocytic infiltration in the lung tissue and the intrabronchiolar and bronchial exudate [22].

### Routine bacteriological culture

Ten microliter of BAL samples collected on D56 was inoculated on Columbia agar supplemented with 5% sheep blood (Oxoid, Hampshire, UK) with a *Staphylococcus pseudintermedius* streak [43]. Plates were incubated in a 5% CO_2_-enriched atmosphere for 48 h at 35 °C to detect the presence of other respiratory bacteria. After 24 and 48 h incubation, all phenotypically different colonies were picked up and identified at species level (score value ≥ 2.000) by matrix-assisted laser desorption/ionization time-of-flight mass spectrometry (MALDI-TOF MS) (MALDI Biotyper, Bruker Daltonics, Bremen, Germany), as previously described [44].

### *M. hyopneumoniae*-specific antibody responses in serum and BAL

The concentration of *M. hyopneumoniae*-specific antibodies in serum was determined using a commercial blocking ELISA (IDEIA^™^ *Mycoplasma hyopneumoniae* EIA kit, Oxoid) according to the manufacturer’s instructions. The presence of *M. hyopneumoniae*-specific immunoglobulin (Ig) G and IgA was measured in serum and BAL samples using an indirect in-house ELISA [45]. Briefly, Tween 20-extracted *M. hyopneumoniae* antigen from the F7.2C strain was used to coat 96-well plates. Serum samples were diluted 1:200 for IgG and 1:10 for IgA, while BAL samples from D15 and D42 were used undiluted and BAL samples from D56 were diluted 1:10 for both IgG and IgA. All samples were tested in duplicate and results were expressed as optical density (OD) values. If the OD difference between both duplicates exceeded 0.05 and was more than 25% of the OD itself, this sample was retested. Samples were considered as positive if the average OD was higher than the cut-off value, which was calculated as the average OD of BAL or serum from animals of Sentinel + 3 times the standard deviation (SD) [23, 46].

### Cytokine immunoassays

The concentration (pg/mL) of interferon γ (IFN-γ), interleukin (IL) 1β (IL-1β), IL-6 and IL-10 was measured in BAL samples using a multiplex immunoassay (Custom Porcine ProcartaPlex Multiplex Immunoassay, ThermoFisher Scientific, Waltham, Massachusetts, USA) as previously described [23]. The concentration of IL-17A (pg/mL) in BAL samples was determined with a commercially available ELISA kit according to the manufacturer’s guidelines (Kingfisher Biotech, Saint Paul, MN, USA) [23]. For both assays, BAL samples were diluted 1:2.

### Digital PCR

Two hundred microliter of each BAL sample was used to extract DNA with a commercial kit (DNeasy® Blood & Tissue kit, Qiagen, Venlo, The Netherlands) following the manufacturer's guidelines. The volume of elution buffer used was 200 µL. A triplex digital PCR (dPCR) assay was created to determine the number of DNA copies of each genetically modified *M. hyopneumoniae* strain and the total *M. hyopneumoniae* DNA copy number.

Specific primers and probes targeting the Tn-insertion sites of each genetically modified *M. hyopneumoniae* strain were developed to measure the number of DNA copies of each genetically modified *M. hyopneumoniae* strain separately (Table 1). The TaqMan^™^ MGB probes for Δ*mmsA* and Δ*mnuA* were purchased from ThermoFisher Scientific and labeled with VIC or FAM, respectively. The total *M. hyopneumoniae* DNA copy number was measured with a previously described dPCR assay targeting the P102 gene of *M. hyopneumoniae* [23, 47]. This double-quenched probe that targets the P102 gene was purchased from IDT (Integrated DNA Technologies, Leuven, Belgium) and labeled with Cy5 as the 5’ fluorophore, TAO as the internal quencher and Iowa Black RQ (IBRQ) as the 3’ quencher. To determine the number of DNA copies of both challenge strains, the copy number of both genetically modified *M. hyopneumoniae* strains was subtracted from the total *M. hyopneumoniae* DNA copy number.

The triplex dPCR was performed with the droplet-based Naica System (Stilla Technologies, Villejuif, France) and 16 reaction cavity Naica Opal chips (Stilla Technologies). The dPCR mixture had a reaction volume of 8 µL and contained 1X PerfeCTa^®^ Multiplex qPCR Toughmix^®^ (VWR International BVBA, Leuven, Belgium), 0.25 µmol/L of each probe, 0.5 µmol/L of each primer, 0.1 µmol/L fluorescein (Merck KGaA, Darmstadt, Germany), 0.4 µL FastDigest EcoRI (ThermoFisher Scientific) and 1.6 µL sample DNA. Highly concentrated samples were diluted using UltraPure^™^ Salmon Sperm DNA Solution (10 µg/mL, ThermoFisher Scientific) as a carrier [23]. During each run, at least 2 non-template controls (NTC) containing reaction mixture and 1.6 µL of 10 µg/mL UltraPure^™^ Salmon Sperm DNA Solution (diluted in elution buffer) were included. The Geode device (Stilla Technologies) was used for thermal cycling. Initial denaturation for 10 min at 95 °C was followed by 40 amplification cycles of denaturation for 15 s at 95 °C and combined annealing/elongation for 30 s at 60 °C. Subsequently, 10 additional amplification cycles of denaturation for 15 s at 95 °C and combined annealing/elongation for 30 s at 56 °C were performed. The Naica Prism3 System (Stilla Technologies) was used for fluorescence readout and a fluorescence spillover compensation matrix was determined and applied to all samples using the CrystalMiner Software (Stilla Technologies). Samples were retested if there were less than 13500 analyzable droplets or oil droplets present in the chamber. The ddpcRquant method was used to apply a baseline correction to account for small shifts in baseline fluorescence [48] and a hard threshold was set at fluorescence 5000 for all three channels for partition classification as shown in Additional file 1 (Blue channel; FAM; Δ*mnuA*), Additional file 2 (Green channel; VIC; Δ*mmsA*) and Additional file 3 (Red channel; Cy5; total *M. hyopneumoniae* DNA). The limit of blank (LOB) and theoretical LOD were calculated using fifty NTCs.

### Cell-mediated immune response

Peripheral blood mononuclear cells (PBMCs) were isolated from fresh, unclotted blood collected immediately before challenge (D28) and at euthanasia (D56) to assess cytokine production by T cells as described before [21, 45]. Briefly, PBMCs were stimulated in vitro with binary ethyleneimine-inactivated *M. hyopneumoniae* F7.2C for 14 h before Brefeldin A was added and cells were incubated for another 4 h. After incubation, cells were stained with anti-CD4 (clone 74-12-4, Southern Biotech, Birmingham, Alabama, USA) and anti-CD8β (clone PG164A, WSU, Pullman, Washington, USA) antibodies and subsequently with their corresponding secondary antibodies anti-mouse IgG2b AlexaFluor 488 (Molecular Probes, Eugene, Oregon, USA) and anti-mouse IgG2a PE-Cy7 (Abcam, Cambridge, UK). After fixation and permeabilization, intracellular cytokine staining was performed using anti-human TNF-α AlexaFluor 647 (clone MAb11, BioLegend, San Diego, California, USA), anti-pig IFN-γ PerCP-Cy5.5 (clone P2G10, Becton–Dickinson, Franklin Lakes, New Jersey, USA) and anti-human IL-17A PE (clone SCPL1362, Becton–Dickinson). Data were acquired with a CytoFLEX flow cytometer (Beckman Coulter, Bea, California, USA). The analyses were performed with the FlowJo^™^ software (Tree Star Inc., Ashland, Oregon, USA). For each animal, samples were stimulated in triplicate cultures and analyzed separately.

### Statistical analyses

Statistical analyses were conducted in SPSS 26 software (IBM, Armonk, New York, USA). The sentinel group was used to verify whether the purchased piglets remained negative for *M. hyopneumoniae* throughout the study, and therefore this group was not included in the statistical analyses. Animals that died prematurely or reached any of the predefined humane endpoints (anorexia, not drinking/serious dehydration, high fever (> 41 °C), lateral decubitus, or severe dyspnea) would have been excluded. In this study, all animals were included in the analyses. Statistical results were considered significant when *P* ≤ 0.05.

For macroscopic lung lesion score, % air, serum and BAL antibodies, BAL cytokine concentration, BAL *M. hyopneumoniae* DNA load and T cell cytokine production, statistical comparisons were made between groups (Δ*mmsA*, Δ*mnuA* and Control) on each sampling day. Normality of residuals and equality of variances were evaluated using Shapiro–Wilk and Levene tests, respectively. For % air, a normal distribution of residuals and equality of variances could be assumed, so this parameter was analyzed using ANOVA with Tukey’s post hoc test to allow pairwise comparisons. Since parametric assumptions were violated for all other abovementioned parameters, a Kruskal–Wallis H-test with Dunn-Bonferroni post hoc test was employed. For these analyses, the abovementioned parameters were included as dependent variable, and group as independent variable.

The median microscopic lung lesion score was analyzed with an ordered logistic regression, with median microscopic lung lesion score as dependent variable and group as independent variable. The effect of both genetically modified *M. hyopneumoniae* strains on the respiratory disease score before and after challenge infection was assessed by a generalized estimating equations procedure with an ordinal logit link function and a subject effect of pig.

## Results

### Growth kinetic of the mutated strains differed from the wild type strain F7.2C

Current commercial *M. hyopneumoniae* vaccines provide only partial protection. In an effort to improve the efficacy of *M. hyopneumoniae* vaccines, a highly virulent *M. hyopneumoniae* strain was attenuated using transposon-mediated disruption of either the *mmsA* or the *mnuA* gene, which might be essential for *M. hyopneumoniae* survival in the host [3, 12]. Since this disruption might affect the growth of *M. hyopneumoniae*, the growth kinetics of these mutated strains were compared to that of the wild type strain F7.2C. As shown in Additional file 4, the growth curves of the wild type strain F7.2C were similar for both repeats, while the growth curves of both genetically modified *M. hyopneumoniae* strains were less similar.

The average ATP peak of the wild type strain was 940 ± 65 pmol ATP/mL and was reached after 5 days. The in vitro growth of Δ*mmsA* resulted in an average ATP peak of 577 ± 48 pmol ATP/mL that was reached after 6–7 days, whereas the average ATP peak of Δ*mnuA* was reached after 6–8 days and was 836 ± 153 pmol ATP/mL. The inoculum used for endotracheal administration of the Δ*mmsA* mutant to piglets contained 583 pmol ATP/mL, and the inoculum of the Δ*mnuA* strain contained 715 pmol ATP/mL. To understand how ATP levels correlated with CCU for these strains, the concentration of both inocula was also determined using the CCU method. After an incubation period of at least 4 weeks, the CCU were measured. The inoculum of Δ*mmsA* contained 1.58 × 10^2^ CCU/mL, while the inoculum of Δ*mnuA* contained 1.58 × 10^6^ CCU/mL. Four weeks after vaccination, the animals were challenged with 5 × 10^8^ CCU/mL of the wild type strain F7.2C.

### *ΔmnuA *reduced coughing of challenged pigs and *M. hyopneumoniae* DNA load in BAL

The newly developed mutated strains were then tested as potential future vaccine candidate strains against *M. hyopneumoniae* infection in pigs. To be able to assess the presence of the Δ*mmsA* and Δ*mnuA* mutants in BAL fluid upon endotracheal administration and to discriminate between the mutant strains and the challenge strains upon challenge infection at day 28, we developed a triplex dPCR assay. This triplex dPCR assay had a LOB of 2, 0 and 0 positive partitions/well for the blue (Δ*mnuA*), green (Δ*mmsA*) and red channel (total *M. hyopneumoniae* DNA), respectively, and a theoretical LOD of 0.76, 0.34 and 0.17 organisms/µL PCR mix for the blue, green and red channel, respectively.

**Figure 2:**
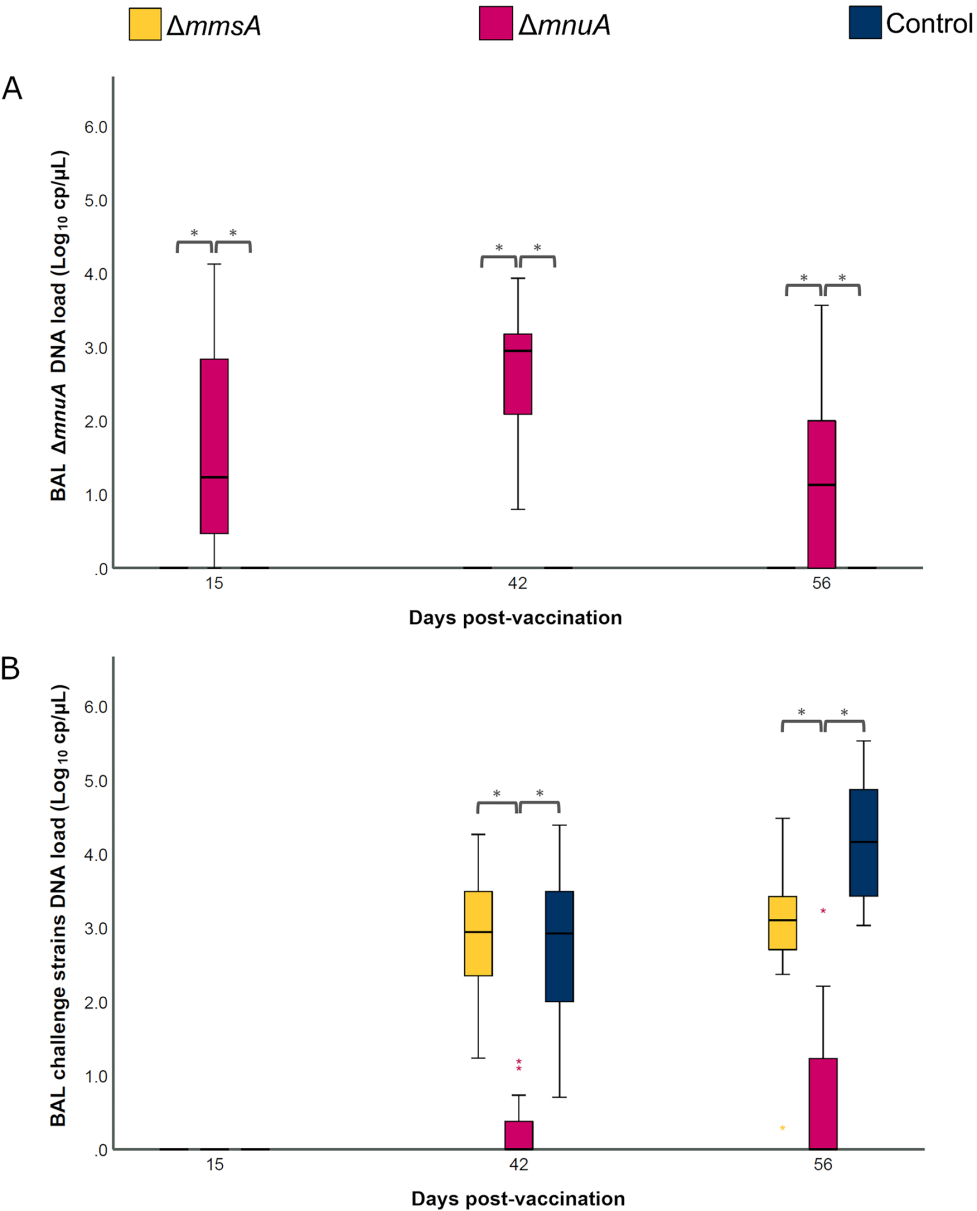
**DNA load of Δ*mnuA* and both *M. hyopneumoniae* challenge strains.** Piglets were vaccinated on D0 with Δ*mmsA*, Δ*mnuA* or physiological saline solution (Control), and animals were challenge infected on D28 and euthanized on D56. The DNA load of Δ*mmsA*, Δ*mnuA* (**A**) and both *M. hyopneumoniae* challenge strains (**B**) was investigated using a digital PCR triplex assay targeting the Tn-insertion sites of both genetically modified *M. hyopneumoniae* strains or the P102 gene. To determine the copy number of both challenge strains, the number of copies of both vaccine candidate strains was subtracted from the total *M. hyopneumoniae* DNA copy number. For each time point, data were analysed using a Kruskal–Wallis H-test with Dunn-Bonferroni post hoc test (*, *p* ≤ 0.05). The center line, box limits and whiskers represent the median, upper and lower quartiles, and 1.5 × interquartile range, respectively.

Animals from the sentinel group remained negative for *M. hyopneumoniae* DNA in BAL throughout the study (data not shown). DNA of the Δ*mmsA* mutant could not be detected in any of the BAL samples collected throughout the experiment (data not shown), while DNA of the Δ*mnuA* mutant could be quantified on each sampling day (Figure 2A). As expected, no DNA of the challenge strains was detected before challenge infection. Two and four weeks after challenge infection, significantly less copies of the DNA from the challenge strains were detected in Δ*mnuA* compared to both Δ*mmsA* and Control (Figure 2B). More specifically, on D42 the average number of DNA copies of the challenge strains was 0.25 Log_10_ copies/µL in Δ*mnuA* as compared to 2.91 and 2.69 Log_10_ copies/µL in Δ*mmsA* and Control, respectively. On D56, the average number of DNA copies of the challenge strains was 2.3–3.5 Log_10_ copies/µL lower in Δ*mnuA* (0.69 Log_10_ copies/µL) as compared to Δ*mmsA* (2.95 Log_10_ copies/µL) and Control (4.19 Log_10_ copies/µL). Moreover, seven animals from Δ*mnuA* did not harbor challenge strain DNA in their BAL on both sampling days (Table 2).

**Table 2:** **Vaccine efficacy parameters**

Parameter	Sentinel	Δ*mmsA*	Δ*mnuA*	Control	*P* value
# animals with challenge strain DNA in BAL collected on D42	0/5	12/12	5/12	12/12	NA
# animals with challenge strain DNA in BAL collected on D56	0/5	12/12	5/12	12/12	NA
Macro LL (0–35)	0.00 (0.00)	0.00 (0.71)	0.00 (0.60)	2.41 (7.98)	0.10
# animals with at least one lobe with macro LL	0/5	5/12	4/12	8/12	NA
Micro LL (0–5)	2.00 (0.50)	2.00 (0.00)	2.00 (0.00)	2.00 (1.40)	0.39
# animals with median micro LL > 2	0/5	2/12	1/12	5/12	NA
% Air	39.00 ± 9.76	37.26 ± 11.07	39.87 ± 10.97	39.28 ± 11.95	0.84

Piglets were vaccinated on D0 with Δ*mmsA*, Δ*mnuA* or physiological saline solution (Control), and animals were challenge infected on D28 and euthanized on D56, after which the number of animals with challenge strain DNA in BAL, the percentage of lung area occupied by air (% Air), macroscopic and microscopic lung lesion score were determined. The sentinel group (Sentinel) was not included in the statistical analyses. % Air is expressed as mean ± standard deviation and ANOVA with a Tukey’s post hoc test was used to investigate significant differences between groups. The macroscopic lung lesion score (macro LL) and the microscopic lung lesion score (micro LL) are expressed as median values (interquartile range). A Kruskal–Wallis H-test with Dunn-Bonferroni post hoc test or an ordered logistic regression was used to explore significant differences between groups for macro LL or micro LL, respectively. Statistical results were considered significant when *p* ≤ 0.05. NA = not analyzed.

**Figure 3:**
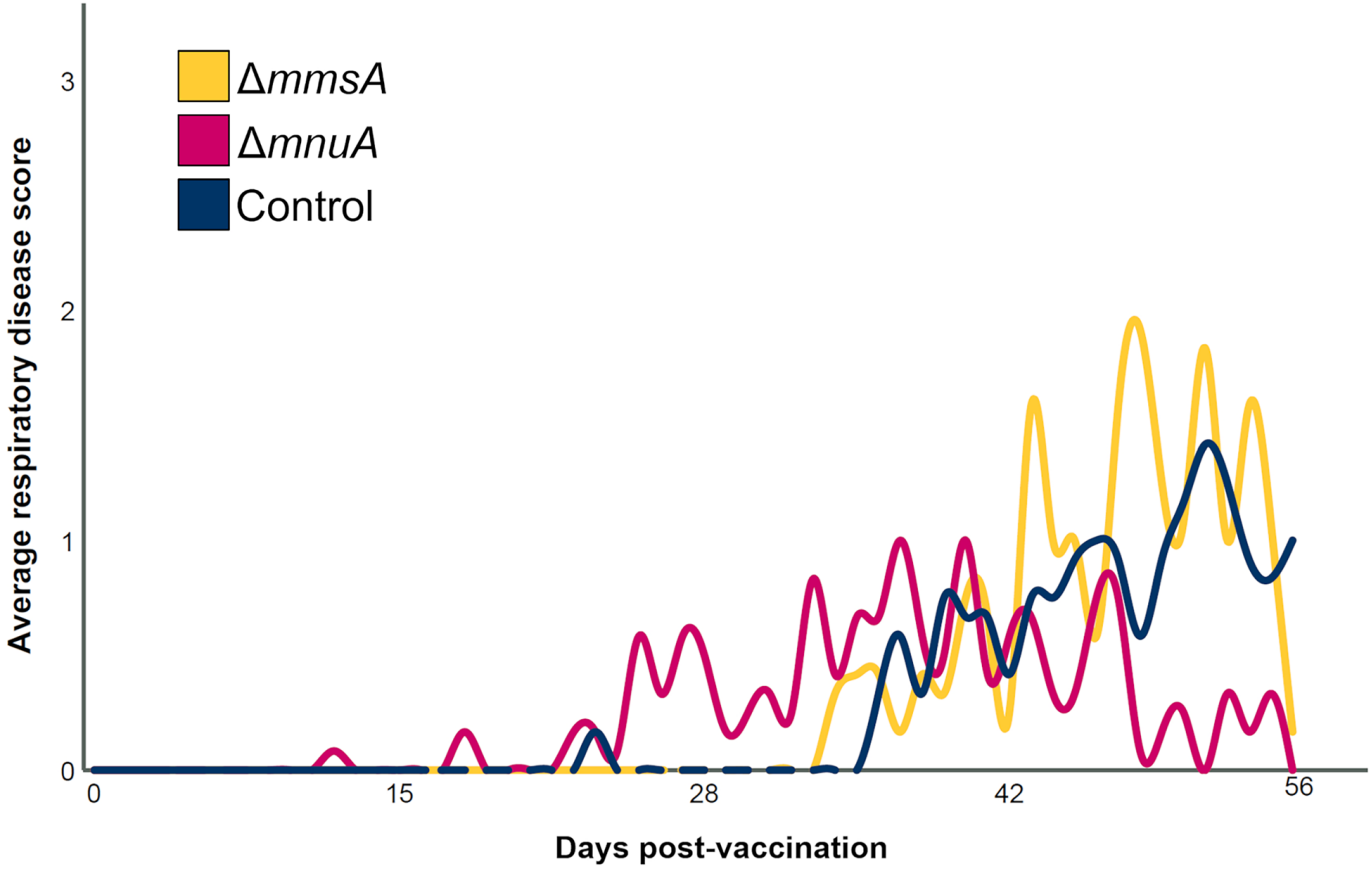
**Average respiratory disease score (RDS) following vaccination and challenge.** Piglets were vaccinated on D0 with Δ*mmsA*, Δ*mnuA* or physiological saline solution (Control), and were challenge infected on D28 and euthanized on D56. For each individual piglet, the severity of coughing was assessed daily by the same investigator using a respiratory disease score (RDS). Significant differences between the groups were determined for two periods, vaccination-challenge and challenge-euthanasia, using a generalized estimating equations procedure with an ordinal logit link function and a subject effect of pig. Statistical results were considered significant when *p* ≤ 0.05. For the period vaccination-challenge, a significantly higher RDS was observed in Δ*mnuA* compared to Δ*mmsA* and Control. For the period challenge-euthanasia, a significantly higher RDS was seen in Δ*mmsA* and Control compared to Δ*mnuA*.

We next sought to determine whether the *M. hyopneumoniae* DNA load in BAL samples was linked with clinical parameters including RDS, macroscopic and microscopic lung lesion score and % air. As shown in Figure 3, endotracheal administration of the Δ*mnuA* mutant resulted in a higher RDS as compared to Δ*mmsA* (*P* < 0.001) and Control (*P* = 0.02), while after challenge infection Δ*mnuA* reduced coughing in animals as evidenced by a lower RDS as compared to Δ*mmsA* (*P* = 0.001) and Control (*P* = 0.02). The RDS was not significantly different between Δ*mmsA* and Control post-challenge. In contrast to the RDS, the other evaluated clinical parameters (macroscopic and microscopic lung lesion score, and % air) did not differ significantly between the groups (Table 2) (Additional file 5).

Taken together, these results showed that the piglets developed a mild cough after administration of the Δ*mnuA* mutant. Furthermore, the animals that received the Δ*mnuA* mutant coughed less after challenge infection as compared to both Δ*mmsA* and Control. This was associated with a lower copy number of challenge strain DNA in BAL samples in Δ*mnuA*.

### Routine bacteriological culture of BAL samples collected at euthanasia

Routine bacteriological culture was performed to detect the presence of respiratory bacteria other than *M. hyopneumoniae*. BAL samples collected at euthanasia from Δ*mmsA* did not contain other respiratory bacteria. In BAL collected on D56 from three animals of Δ*mnuA* and two animals of Control, a limited number of colonies of different bacterial species were detected and therefore not considered as clinically relevant.

### *M. hyopneumoniae*-specific antibody responses are induced in serum and BAL of vaccinated animals

**Figure 4:**
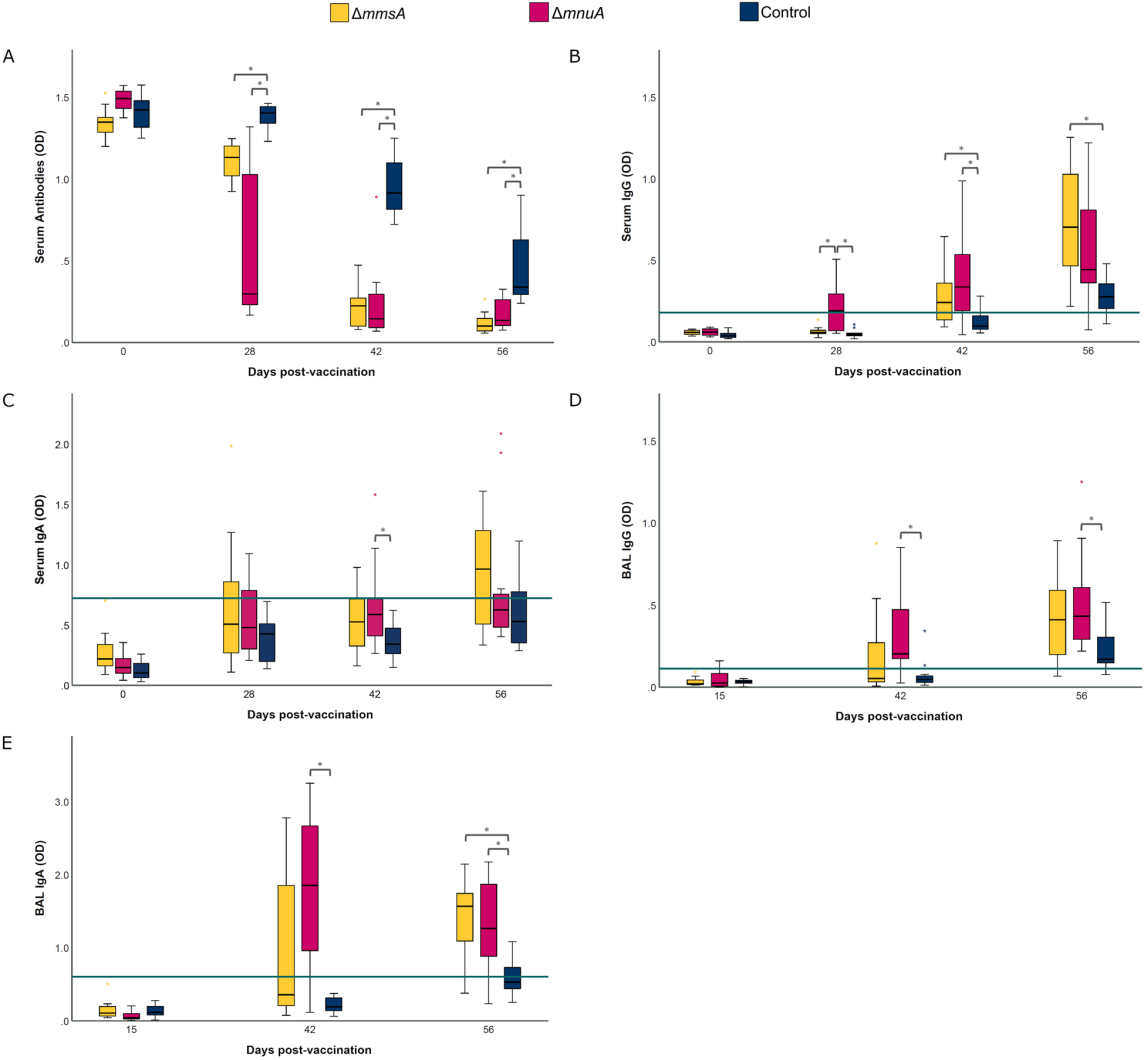
**Serum and BAL antibody levels following vaccination and challenge.** Piglets were vaccinated on D0 with Δ*mmsA*, Δ*mnuA* or physiological saline solution (Control), and animals were challenge infected on D28 and euthanized on D56. Antibody levels against *Mycoplasma hyopneumoniae* were measured in serum with a commercial blocking ELISA (**A**). In a blocking ELISA, a lower OD level corresponds with a higher antibody level. An indirect ELISA was used to determine the concentration of serum IgG (**B**), serum IgA (**C**), BAL IgG (**D**) and BAL IgA (**E**). For *M. hyopneumoniae*-specific IgG and IgA, serum and BAL samples were considered positive if the average OD value was higher than the cut-off value (green horizontal line). For each time point, significance was calculated using a Kruskal–Wallis H-test with Dunn-Bonferroni post hoc test (*, *p* ≤ 0.05). OD = optical density. The center line, box limits and whiskers represent the median, upper and lower quartiles, and 1.5 × interquartile range, respectively.

To explore the immune responses elicited by vaccination and challenge infection, we assessed the *M. hyopneumoniae*-specific antibody responses by ELISA in serum and BAL. As expected, animals from Sentinel remained negative for *M. hyopneumoniae*-specific serum antibodies throughout the study (data not shown). As shown in Figure 4A, all animals from all groups were seronegative for *M. hyopneumoniae*-specific antibodies before vaccination. Four weeks after vaccination (D28), the majority of animals in Δ*mnuA* appeared to have seroconverted in contrast to the animals of Δ*mmsA*. Two weeks after challenge, even though most animals from Control and all animals from Δ*mmsA* and Δ*mnuA* had seroconverted, a significantly higher level of serum antibodies was observed in both vaccinated groups as compared to Control. Four weeks after challenge, all animals from Δ*mmsA*, Δ*mnuA* and Control had *M. hyopneumoniae*-specific serum antibodies, but the concentration of these antibodies was significantly higher in Δ*mmsA* and Δ*mnuA* as compared to Control.

An in-house ELISA was used to gain more insights in the antibody class that was induced in serum by both genetically modified *M. hyopneumoniae* strains. While *M. hyopneumoniae*-specific serum IgG and IgA levels were below the cut-off value on D0 in all groups (Figures 4B, C), Δ*mnuA* elicited higher serum IgG responses as compared to Δ*mmsA* and Control on D28. Two weeks after challenge, significantly higher serum IgG levels were detected in both vaccinated groups. Only in Δ*mnuA*, significantly higher serum IgA levels could be observed on D42, but this was mainly due to high serum IgA levels in two animals. At euthanasia, serum IgG levels were significantly higher in Δ*mmsA* compared to Control. Given the endotracheal administration of the genetically modified *M. hyopneumoniae* strains, we also evaluated local antibody responses. As shown in Figures 4D, E, at 2 weeks post-vaccination, BAL IgG and IgA levels remained below the threshold in all groups. Nevertheless, upon challenge infection, BAL IgG and IgA levels were significantly higher in Δ*mnuA* as compared to Control, while only on D56 BAL IgA levels were also significantly higher in Δ*mmsA* as compared to Control.

### Vaccinated animals produced less local pro-inflammatory cytokines after challenge infection

**Figure 5:**
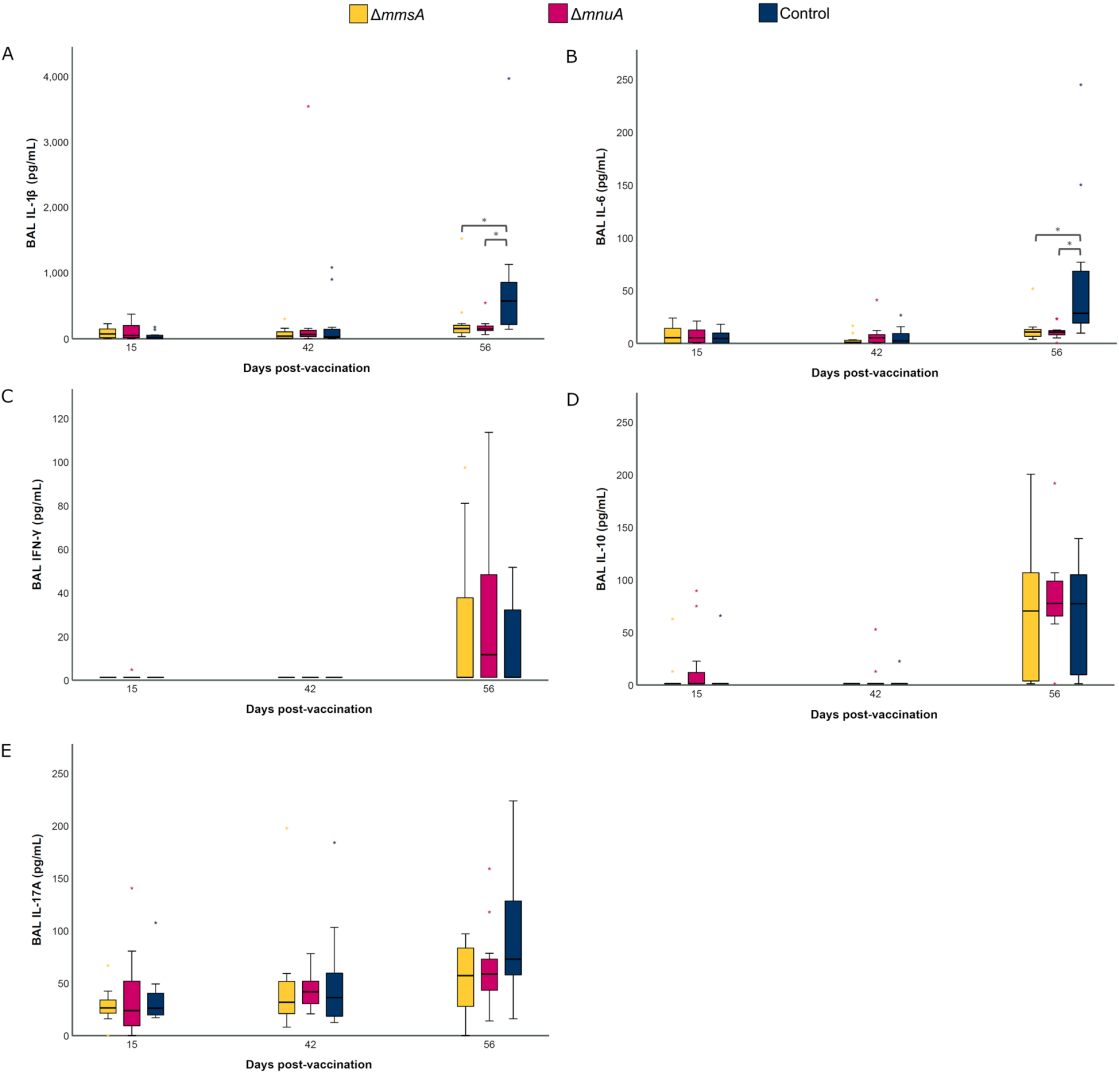
**BAL cytokine levels following vaccination and challenge.** Piglets were vaccinated on D0 with Δ*mmsA*, Δ*mnuA* or physiological saline solution (Control), and animals were challenge infected on D28 and euthanized on D56. The concentration of IL-1β (**A**), IL-6 (**B**), IFN-γ (**C**) and IL-10 (**D**) was determined using a multiplex immunoassay, while the concentration of IL-17A (**E**) was determined with a commercial ELISA. For each time point, data were analysed using a Kruskal–Wallis H-test with Dunn-Bonferroni post hoc test (*, *p* ≤ 0.05). OD = optical density. The center line, box limits and whiskers represent the median, upper and lower quartiles, and 1.5 × interquartile range, respectively.

Since previous research by our group and others has revealed that vaccination can alter the production of local cytokines after challenge infection [21–23, 49], we measured the cytokine concentration of different pro- and anti-inflammatory cytokines in BAL to study the effect of both genetically modified *M. hyopneumoniae* strains on infection-induced cytokines. Figure 5A, B shows that four weeks after challenge, a lower concentration of the pro-inflammatory cytokines IL-1β and IL-6 could be detected in BAL samples from both vaccinated groups as compared to Control. However, both genetically modified *M. hyopneumoniae* strains did not affect the concentration of IFN-γ, IL-10 or IL-17A at any time point during the experiment (Figures 5C–E).

### Cytokine production by CD8^+^ T cells is altered after vaccination and challenge infection

**Figure 6:**
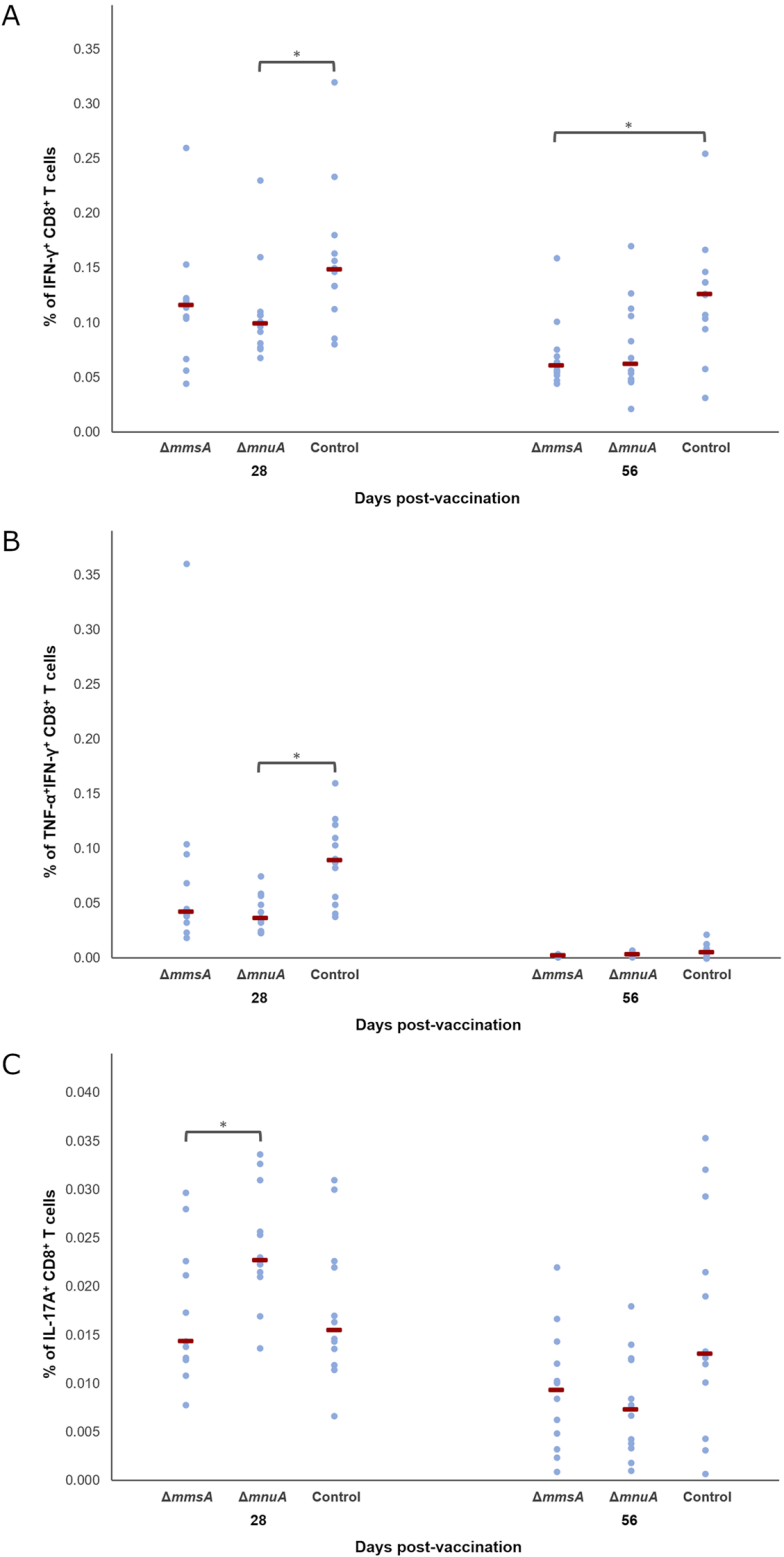
**Percentage of *M. hyopneumoniae*-specific cytokine-producing T cell subsets.** Piglets were vaccinated on D0 with Δ*mmsA*, Δ*mnuA* or physiological saline solution (Control), and animals were challenge infected on D28 and euthanized on D56. The percentage of *M. hyopneumoniae*‑specific IFN-γ^+^ CD8^+^ (**A**), TNF-α^+^IFN-γ^+^ CD8^+^ (**B**) and IL-17A^+^ CD8^+^ (**C**) T cells was determined using a recall assay and flow cytometry. For each time point, data were analysed using a Kruskal–Wallis H-test with Dunn-Bonferroni post hoc test (*, *p* ≤ 0.05). Median values for each group are shown (red line).

To assess activation of cell-mediated immunity by both genetically modified *M. hyopneumoniae* strains, we measured the cytokine production by circulating CD4^+^ and CD8^+^ T cells. We did not detect statistically significant differences between the groups four weeks after vaccination (D28) or four weeks after challenge infection (D56) for the percentage of cytokine-producing CD4^+^ or TNF-α^+^ CD8^+^ T cells (data not shown), but the percentage of IFN-γ^+^, TNF-α^+^IFN-γ^+^ or IL-17A^+^ CD8^+^ T cells differed significantly after vaccination and/or challenge infection (Figure 6). Four weeks after vaccination, significantly lower percentages of IFN-γ^+^ and TNF-α^+^IFN-γ^+^ CD8^+^ T cells were observed in Δ*mnuA* as compared to Control. Furthermore, the percentage of IL-17A^+^ CD8^+^ T cells was significantly higher in Δ*mnuA* as compared to Δ*mmsA*. After challenge infection, no significant differences were seen between the groups in the percentage of TNF-α^+^IFN-γ^+^ or IL-17A^+^ CD8^+^ T cells, but the percentage of IFN-γ^+^ CD8^+^ T cells turned out to be significantly lower in Δ*mmsA* as compared to Control.

## Discussion

Live vaccines are generally more immunogenic than inactivated ones [16]. Nevertheless, only two attenuated *M. hyopneumoniae* vaccines have been licensed thus far [18–20]. In a search for *M. hyopneumoniae* vaccines that might provide better protection against clinical disease, we developed two genetically modified *M. hyopneumoniae* strains based on a highly virulent *M. hyopneumoniae* field strain which were evaluated in an experimental challenge infection study to investigate their efficacy. Ideally, such new vaccines do not only reduce clinical signs and economic losses, but also decrease or even avoid pathogen transmission and colonization. Even though promising results were observed after administration of Δ*mnuA*, more research is required to further explore its potential to reduce or even inhibit colonization and transmission of *M. hyopneumoniae* in pigs.

The difficulty in culturing *M. hyopneumoniae*, but also the risk of reversion to virulence might be one of the reasons why only two attenuated *M. hyopneumoniae* vaccines have been licensed thus far. One attenuated vaccine used in Mexico (Vaxsafe® MHP, Avimex) is a thermosensitive mutant of *M. hyopneumoniae* which is administered intranasally [19, 20]. Little information is available on the immunogenicity and efficacy of this Mexican attenuated vaccine. The other attenuated vaccine is licensed in China and is derived from a virulent Mhp 168 parent strain which was isolated in 1974 in China and has been gradually attenuated by continuous passage through modified Friis medium [18, 50]. The Chinese vaccine is mostly administered intrapulmonary [50], but it has also been administered as an aerosol, intranasally or intramuscularly [18, 28, 51, 52]. Intramuscular administration of the Chinese attenuated vaccine in combination with different adjuvants seemed to evoke a *M. hyopneumoniae*-specific serum antibody response [51], local *M. hyopneumoniae*-specific secretory IgA [18, 28] as well as an elevated lymphocyte proliferation response against *M. hyopneumoniae* antigen [50–52]. For intranasal and aerosol administration of the Chinese vaccine, no challenge studies have been performed, but intraperitoneal [50] or intramuscular [51, 52] vaccination resulted in a significant reduction of the macroscopic lung lesion score compared to a non-vaccinated, challenged control group. However, the macroscopic lung lesion score, which varied between 0 (no *Mycoplasm*a-like lung lesions) and 28 (> 75% of the entire lung affected), was not reduced to 0 (the score was at least 3.57 for animals of the vaccinated groups), thus a full protection against a *M. hyopneumoniae* infection could not yet be obtained [50–52].

Even though more studies are required to confirm our results, Δ*mnuA* seems to be a good potential candidate for future attenuated *M. hyopneumoniae* vaccines. Most commercially available vaccines are based on the J strain, which was isolated in 1963 from a field outbreak in sows showing mild EP [33]. In contrast, the genetically modified *M. hyopneumoniae* strains in our study are based on a more recently isolated high virulence *M. hyopneumoniae* strain (F7.2C) [34]. The J strain was originally a virulent strain but underwent continuous passages in vitro, similar to the Mhp 168 strain. A proteomic study comparing *M. hyopneumoniae* strains 232 and J revealed that after multiple in vitro passages, the attenuated J strain modified its focus to metabolism to profit more from the rich culture medium and the ability to infect host cells became less important resulting in a lower expression of adhesion-related genes as compared to the high virulence strain 232 [53]. Furthermore, for several bacterial species, including mycoplasmas, it has been reported that the amount of capsular polysaccharides is a major factor for virulence [54], which decreases substantially with in vitro passages [55]. Therefore, it is questionable whether *M. hyopneumoniae* strains that underwent numerous continuous passages in vitro have similar characteristics as the *M. hyopneumoniae* strains that circulate on farms today, and whether such strains should be used for contemporary *M. hyopneumoniae* vaccines.

In this study, the growth of both genetically modified *M. hyopneumoniae* strains was measured using a fast and time efficient ATP assay and a time consuming CCU technique [37]. While the ATP assay uses an enzymatic reaction to measure cellular ATP, the CCU method detects a color shift in the culture medium due to acidification during growth [37, 56]. In Δ*mnuA*, the *mnuA* gene in the *M. hyopneumoniae* strain F7.2C was disrupted, and in Δ*mmsA*, the *mmsA* gene (which is involved in the myo-inositol pathway) was disrupted. *Mycoplasma hyopneumoniae* strains take up myo-inositol when grown in glucose-rich Friis medium, although this myo-inositol uptake is not essential for in vitro growth of *M. hyopneumoniae* [3]. Interestingly, *M. hyopneumoniae* produces high concentrations of acetate in Friis medium, which drop in the absence of myo-inositol [3]. The accumulation of acetate is known to acidify the medium [57]. Thus, it is possible that disrupting the *mmsA* gene in Δ*mmsA* resulted in the inability of this strain to metabolize myo-inositol, which might lead to a lower acetate production and thereby a slower decrease in pH. Hence, this could explain the discrepancy between the ATP and CCU measurement of Δ*mmsA*. Alternatively, disrupting the *mmsA* gene might lead to accumulation of cellular ATP in the absence of bacterial growth.

It is remarkable that a single endotracheal vaccination with Δ*mnuA* leads to a peak in the number of copies of its DNA in BAL six weeks after vaccination and the recovery of its DNA until euthanasia. On the other hand, DNA from Δ*mmsA* was not recovered in BAL during the experiment. Therefore, either the dose given was too low (assuming no bacterial growth) or disrupting the *mmsA* gene resulted in a too strong attenuation of this genetically modified *M. hyopneumoniae* strain, which was not able to colonize the lungs. Either way, the concentration of Δ*mmsA* might have been too low to provide a (good) level of protection. A higher concentration of Δ*mmsA* might lead to colonization of the lungs, however, this genetically modified *M. hyopneumoniae* strain might not be an ideal potential future vaccine candidate strain as we were not able to obtain a high concentration of this strain when using the normal growth conditions of *M. hyopneumoniae*, which might complicate a reliable vaccine production at a larger scale.

After vaccination with Δ*mnuA*, the piglets developed a mild cough. Coughing after vaccination might to a certain degree be seen as an adverse effect. However, the balance between sufficient colonization of the lungs to trigger (protective) immune responses after a single dose vaccination while avoiding long lasting effects of *M. hyopneumoniae* vaccination on animal wellbeing, general health and economic consequences is very delicate. Further research, e.g. exploring different vaccination routes, focusing on the role of adjuvants or vaccination dose, responses in animals that are not challenge infected as well as a possible reversion of the mutation in vitro and in vivo is needed to optimize this genetically modified *M. hyopneumoniae* strain to serve as a potential attenuated vaccine candidate strain in the future. Furthermore, it is equally important that these potential future *M. hyopneumoniae* vaccine candidate strains are tested further (e.g. using different volumes, other administration routes…) and under field conditions, including vaccination at a younger age, to determine their ability to protect animals under field conditions.

All animals from the control group were positive for the *M. hyopneumoniae* challenge strain in BAL and seroconverted four weeks after challenge infection. Nevertheless, in the current study, the macroscopic lung lesion score of the control group (2.41) was low as compared to previous studies using the same infection model. In these studies, the macroscopic lung lesion score of the control group ranged between 4.14 ± 2.30 and 7.57 ± 5.18 [21–23, 40, 58]. Since we followed the same challenge infection procedure and the *M. hyopneumoniae* DNA load was comparable as in those previous studies, we assume that the lower macroscopic lung lesion score could be attributed to seasonal differences or differences between the individual animals. Nevertheless, it should be noted that the median macroscopic and microscopic lung lesion scores of both vaccinated groups were very low (see Table 2).

Δ*mnuA* was able to significantly reduce the respiratory disease score post-challenge and the copy number of challenge strain DNA. It is noteworthy that in previous experimental studies in pigs using experimental and commercial *M. hyopneumoniae* bacterins and subunit vaccines, such a strong reduction of *M. hyopneumoniae* challenge strain DNA (3–4 logs difference) has not been detected yet [17, 21–23, 59–62]. One of the mechanisms by which live vaccines can provide some degree of protection against infection with the wild type pathogen is the phenomenon of colonization-inhibition. Colonization-inhibition is usually more important shortly after vaccination (even within a few hours after vaccination, as investigated in-depth in *Salmonella* for example [63]). In this study, animals were challenge infected 4 weeks after vaccination. Since the period between vaccination and challenge infection was rather long, it is unlikely that the effect we observed in the Δ*mnuA* group can be attributed solely to the colonization-inhibition phenomenon. Also the effect of Δ*mnuA* on the immune responses is an important factor that could explain the significantly lower RDS in Δ*mnuA* after challenge infection. Nevertheless, additional studies with these genetically modified *M. hyopneumoniae* strains, including longer vaccination-challenge intervals, are required.

We observed a significantly higher serum IgG concentration in Δ*mnuA* four weeks after vaccination. After challenge infection, both vaccinated groups showed significantly higher serum IgG and BAL IgA levels as compared to the control group, and animals from Δ*mnuA* also possessed a higher concentration of BAL IgG. The role of antibodies in protection against a *M. hyopneumoniae* infection remains a matter of debate [25, 32, 64, 65]. Here, we showed that Δ*mnuA* induced antibody responses and that this resulted in higher IgA and IgG in BAL after challenge infection, a lower number of copies of challenge strain DNA and a lower RDS.

Besides an effect on the local antibodies, vaccination can also affect the production of inflammatory cytokines after challenge infection [21–23, 32]. While local pro-inflammatory cytokines can help clear a *M. hyopneumoniae* infection, these responses can also lead to the development of lung lesions [66]. In contrast to parenteral vaccines, mucosal vaccines induce a strong local immune response, making the latter route of administration the preferred vaccination route [16, 35]. Interestingly, despite the endotracheal administration of our genetically modified *M. hyopneumoniae* strains, and the presence of Δ*mnuA* DNA in BAL, a local pro-inflammatory cytokine response could not be detected after vaccination, which could indicate that Δ*mnuA* will probably not elicit major lesions shortly after vaccination. Nevertheless, to assess the safety of genetically modified *M. hyopneumoniae* strains that might serve as potential future attenuated vaccine candidates, additional studies, including an administration of 10 times the normal dosage or a 5-generation feedback challenge, should be conducted. After challenge, we have detected a lower pro-inflammatory cytokine response in both vaccinated groups, a finding that has also been reported in other *M. hyopneumoniae* vaccine studies [22, 23, 32].

Although it is believed that cell-mediated immune responses are important to protect pigs against infection with *M. hyopneumoniae* [29, 67], only few studies have been conducted to evaluate the effect of vaccination on the activation of different T cell subsets by measuring their intracellular cytokine production [21, 23, 45, 46]. It has been suggested that multifunctional T cells, which are capable of producing two or even three cytokines simultaneously, play a central role in protective immunity in humans [68, 69], mice [70, 71], and also pigs [72]. Unfortunately, only few studies on the functional role of porcine CD8^+^ T cells have been conducted [73]. Thus far, porcine CD8β^+^ T cells are considered to have a cytolytic function and to be involved in protective immunity against viral infections [74, 75]. In addition, porcine CD8β^+^ T cells are potent producers of effector cytokines like IFN-γ and TNF-α, especially in non-lymphatic organs. Lung tissue appeared to be strongly enriched with IFN-γ single-producing as well as IFN-γ and TNF-α co-producing CD8β^+^ T cells [75]. Indeed, high numbers of antigen-specific IFN-γ, TNF-α, and IFN-γ and TNF-α co-producing CD8^+^ T cells have been found in lung tissue and BAL collected from animals experimentally infected with swine influenza virus type A [76, 77]. In contrast, a much lower proportion of these cytokine-producing CD8^+^ T cells were detected in blood, suggesting homing of effector CD8^+^ T cells to the lung. In mice, it has been proposed that specialized CD4^+^ and CD8^+^ T cells are compartmentalized due to migration of different subsets to specific sites [78]. However, whether migration of subsets is at the basis for the suggested homing potential of CD8^+^ T cells in pigs remains to be established [76]. In our study, we did not observe an effect of endotracheal administration of our genetically modified *M. hyopneumoniae* strains on the cytokine production by blood CD4^+^ T cells. In contrast, we did notice that animals vaccinated with Δ*mnuA* had significantly less IFN-γ^+^ and TNF-α^+^IFN-γ^+^ CD8β^+^ T cells in their blood post-vaccination compared to control animals. It is tempting to speculate that this lower percentage of cytokine-producing blood CD8^+^ T cells might be the result of migration of these effector CD8^+^ T cells to the lung, as has been proposed in mice. Surprisingly, in one of our previous studies we observed the opposite, namely a significantly higher percentage of TNF-α^+^IFN-γ^+^ CD8^+^ T cells in blood collected from animals four weeks after they were vaccinated intramuscularly with different experimental *M. hyopneumoniae* bacterins [45]. It is difficult to pinpoint the exact reason for this apparently contradictory finding, but we believe that the administration route (endotracheal vs. intramuscular) or the different vaccine type used (attenuated vaccine vs. bacterin) might play a role in the elicited cell-mediated immune responses upon vaccination. Nevertheless, it is important to further examine the effect of *M. hyopneumoniae* vaccination on cell-mediated immune responses in order to elucidate the mechanisms of protection, as well as explore new *M. hyopneumoniae* vaccines like attenuated vaccines and alternative vaccine administration routes to design next generation vaccines that provide a high level of protection against clinical disease and can block transmission.

In conclusion, a single endotracheal administration of Δ*mnuA* seemed to provide a good level of protection against clinical disease, as evidenced by the lower RDS post-challenge and the low copy number of *M. hyopneumoniae* challenge strain DNA. Furthermore, both genetically modified *M. hyopneumoniae* strains evoked an increase in serum antibodies, as well as local immune responses post-challenge. Even though the results of this exploratory study are especially promising for Δ*mnuA*, more in-dept research is required to validate these observed effects, to characterize its safety aspects and to optimize its administration route, dose and formulation.

## Supplementary Information

**Additional file 1. Threshold at fluorescence 5000 for blue channel to determine partition classification in digital PCR assay. **BAL samples were analyzed using a digital PCR assay targeting the Tn-insertion site of Δ*mnuA*. After thermal cycling, fluorescence readout and a fluorescence spillover compensation matrix, small discrepancies in baseline fluorescence were corrected and a hard threshold was set at fluorescence 5000 to allow partition classification.

**Additional file 2. Threshold at fluorescence 5000 for green channel to determine partition classification in digital PCR assay. **BAL samples were analyzed using a digital PCR assay targeting the Tn-insertion site of Δ*mmsA*. After thermal cycling, fluorescence readout and a fluorescence spillover compensation matrix, small discrepancies in baseline fluorescence were corrected and a hard threshold was set at fluorescence 5000 to allow partition classification.

**Additional file 3. Threshold at fluorescence 5000 for red channel to determine partition classification in digital PCR assay. **BAL samples were analyzed using a digital PCR assay targeting the P102 gene of *M. hyopneumoniae*. After thermal cycling, fluorescence readout and a fluorescence spillover compensation matrix, small discrepancies in baseline fluorescence were corrected and a hard threshold was set at fluorescence 5000 to allow partition classification.

**Additional file 4. Growth tests of both genetically modified *M. hyopneumoniae* strains. **Two growth curves are presented for the wild type high virulence *M. hyopneumoniae* strain F7.2C and three growth curves are presented for each genetically modified *M. hyopneumoniae* strain. The ATPwas measured in a 10 mL culture during several days. The growth of both genetically modified *M. hyopneumoniae* strains was less predictable as compared to the non-attenuated F7.2C strain.

**Additional file 5. Macroscopic lung lesion score. **Piglets were vaccinated on D0 with Δ*mmsA*, Δ*mnuA*or physiological saline solution, and animals were challenge infected on D28 and euthanized on D56, after which the macroscopic lung lesion score was determined for each pig. The macroscopic lung lesion scoreis shown in a scatter plot. The median value of each group is also mentioned in Table 2.

## Data Availability

The original contributions presented in the study are included in the article or supplementary material. The datasets used and/or analysed during the current study are available from the corresponding author on reasonable request.
