# Supplementary figures and images for: Efficacy of genetically modified Mycoplasma hyopneumoniae strains and their effect on local and cell-mediated immune responses in pigs
Source: Vet Res. 2025 Nov 17;56:217. doi: 10.1186/s13567-025-01653-2 (PMC12625135; doi:10.1186/s13567-025-01653-2)

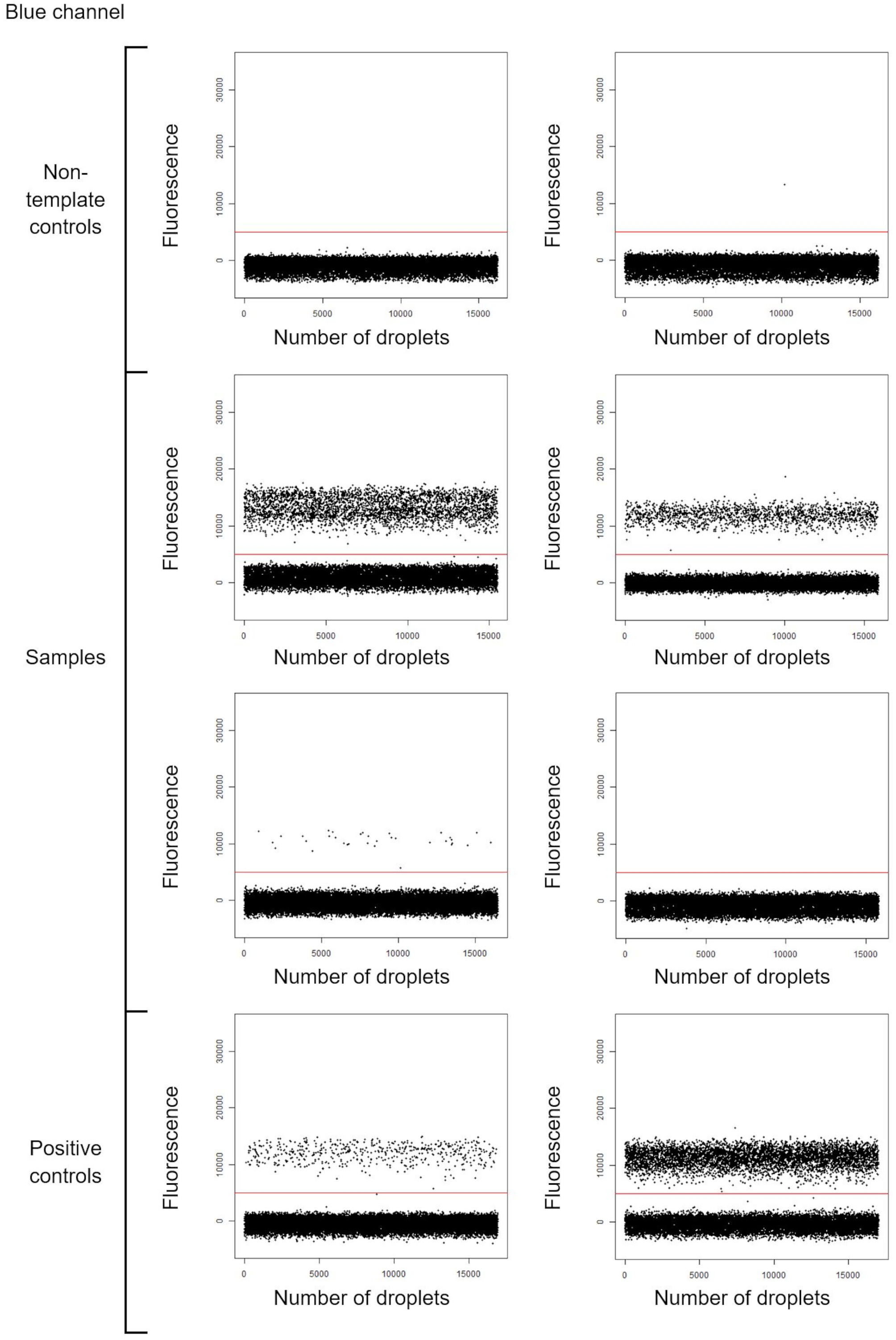

Supplement: Supplementary file 1 — Additional file 1. Threshold at fluorescence 5000 for blue channel to determine partition classification in digital PCR assay. BAL samples were analyzed using a digital PCR assay targeting the Tn-insertion site of ΔmnuA. After thermal cycling, fluorescence readout and a fluorescence spillover compensation matrix, small discrepancies in baseline fluorescence were corrected and a hard threshold was set at fluorescence 5000 to allow partition classification. [file 13567_2025_1653_MOESM1_ESM.jpg]

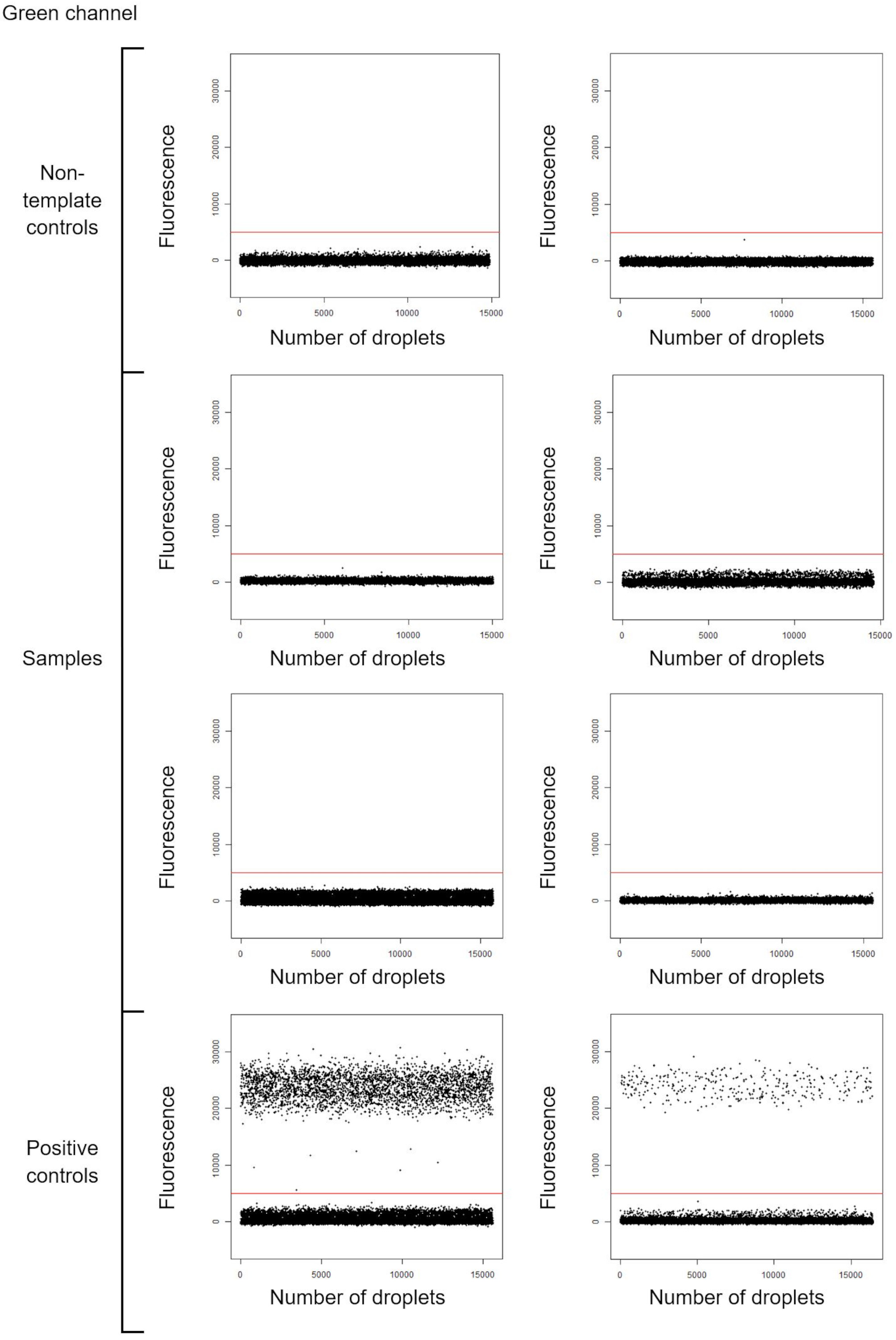

Supplement: Supplementary file 2 — Additional file 2. Threshold at fluorescence 5000 for green channel to determine partition classification in digital PCR assay. BAL samples were analyzed using a digital PCR assay targeting the Tn-insertion site of ΔmmsA. After thermal cycling, fluorescence readout and a fluorescence spillover compensation matrix, small discrepancies in baseline fluorescence were corrected and a hard threshold was set at fluorescence 5000 to allow partition classification. [file 13567_2025_1653_MOESM2_ESM.jpg]

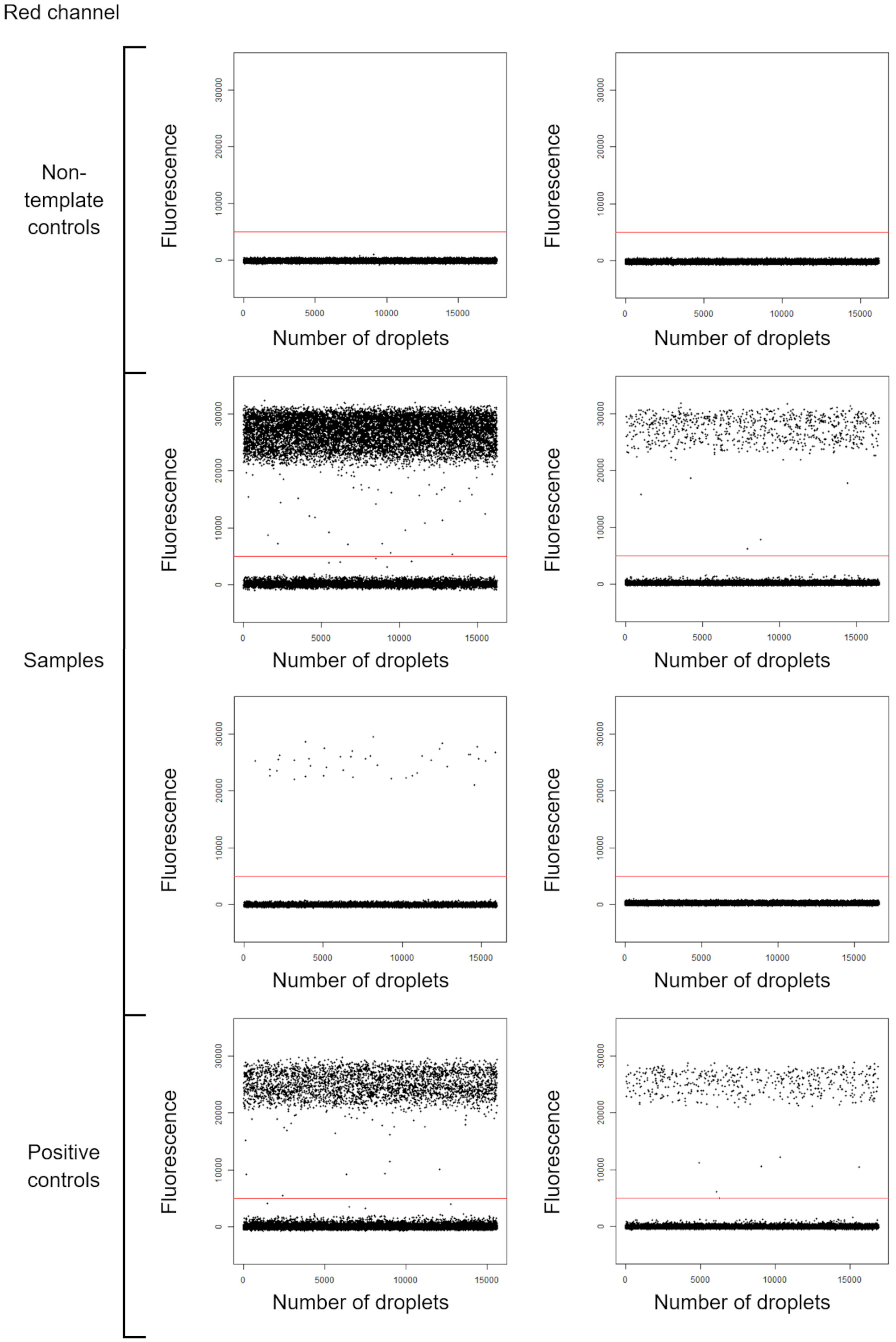

Supplement: Supplementary file 3 — Additional file 3. Threshold at fluorescence 5000 for red channel to determine partition classification in digital PCR assay. BAL samples were analyzed using a digital PCR assay targeting the P102 gene of M. hyopneumoniae. After thermal cycling, fluorescence readout and a fluorescence spillover compensation matrix, small discrepancies in baseline fluorescence were corrected and a hard threshold was set at fluorescence 5000 to allow partition classification. [file 13567_2025_1653_MOESM3_ESM.jpg]

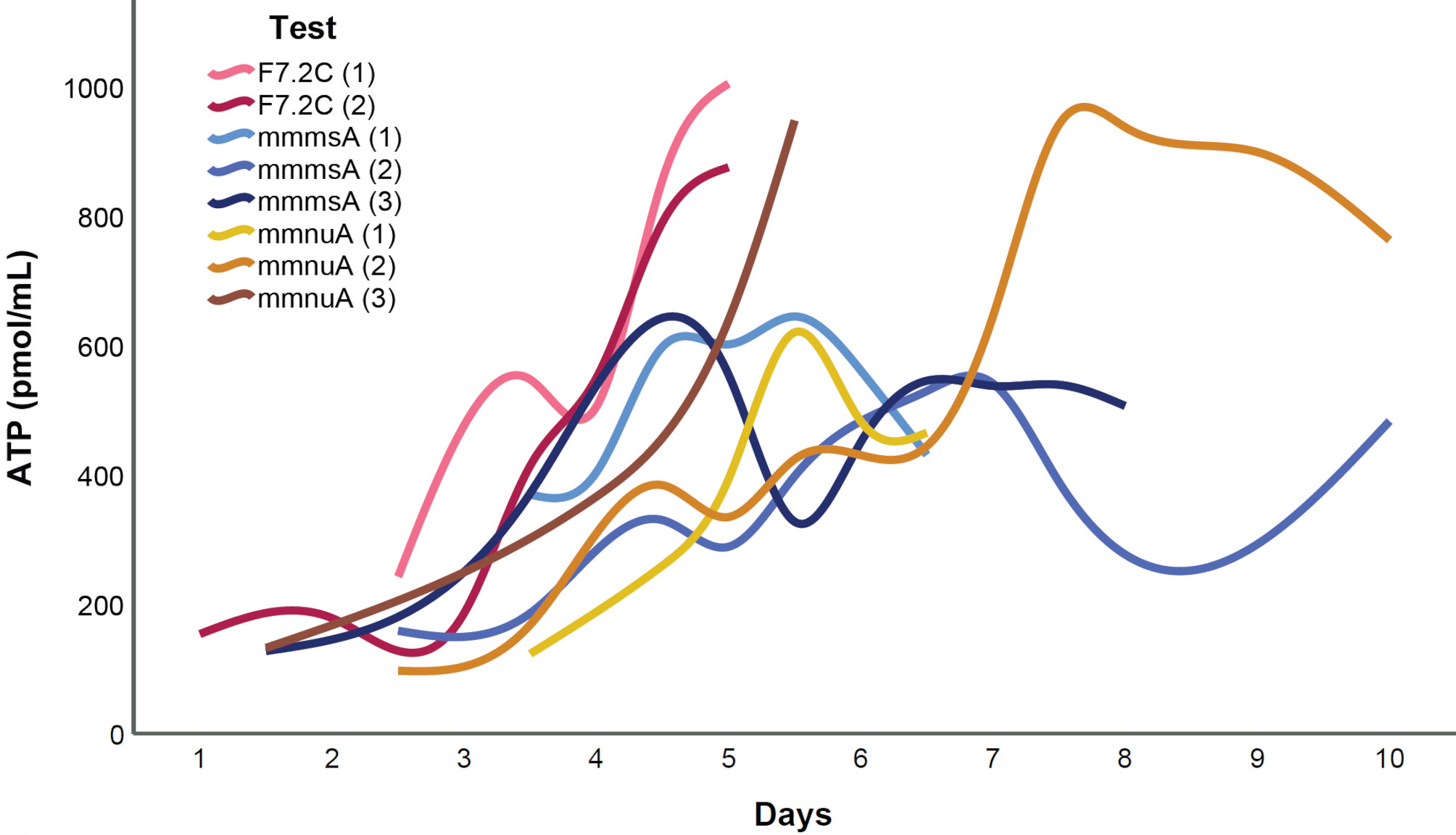

Supplement: Supplementary file 4 — Additional file 4. Growth tests of both genetically modified M. hyopneumoniae strains. Two growth curves are presented for the wild type high virulence M. hyopneumoniae strain F7.2C and three growth curves are presented for each genetically modified M. hyopneumoniae strain. The ATPwas measured in a 10 mL culture during several days. The growth of both genetically modified M. hyopneumoniae strains was less predictable as compared to the non-attenuated F7.2C strain. [file 13567_2025_1653_MOESM4_ESM.jpg]

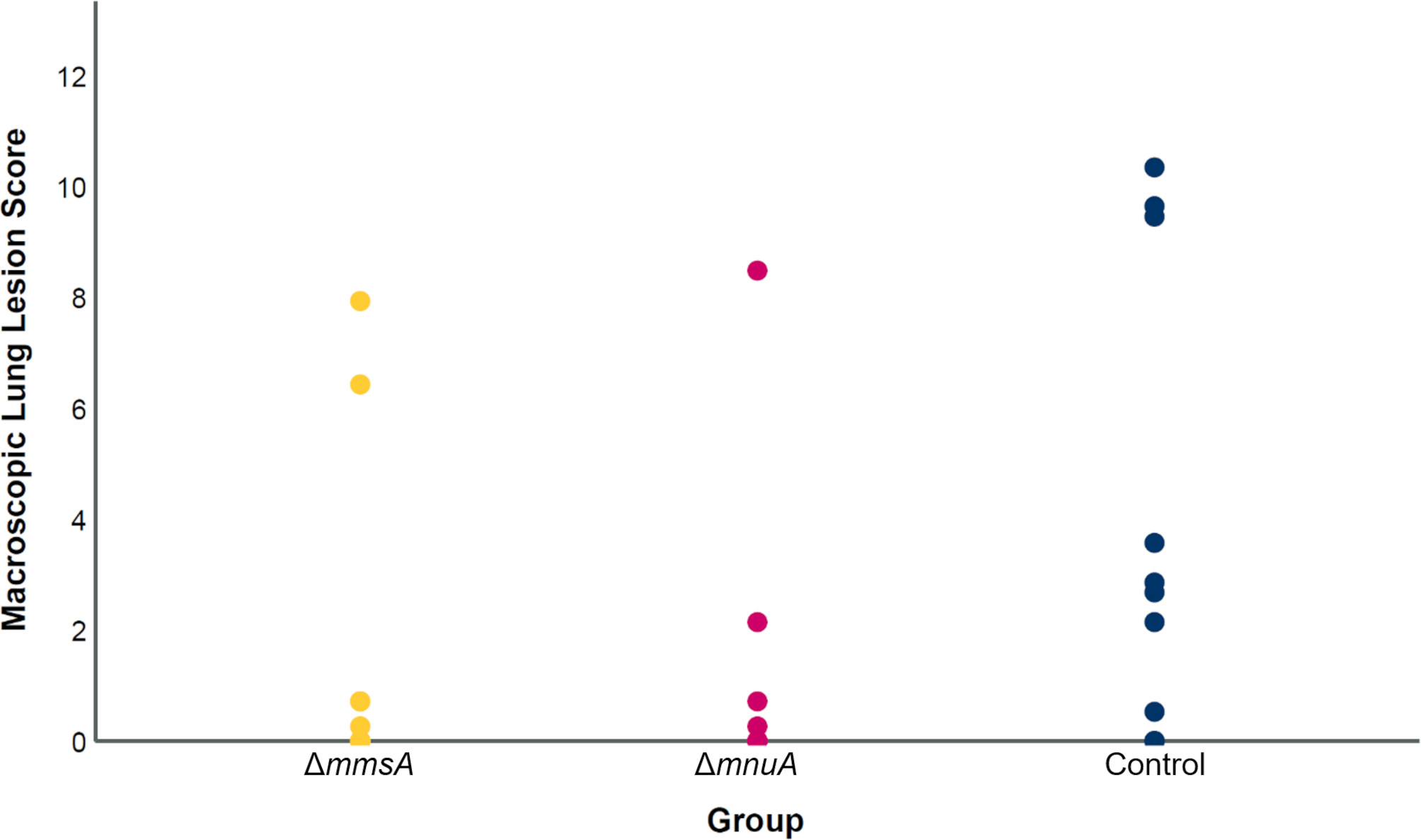

Supplement: Supplementary file 5 — Additional file 5. Macroscopic lung lesion score. Piglets were vaccinated on D0 with ΔmmsA, ΔmnuAor physiological saline solution, and animals were challenge infected on D28 and euthanized on D56, after which the macroscopic lung lesion score was determined for each pig. The macroscopic lung lesion scoreis shown in a scatter plot. The median value of each group is also mentioned in Table 2. [file 13567_2025_1653_MOESM5_ESM.jpg]
